# Supplementary material for: IMARA: A mother-daughter group randomized controlled trial to reduce sexually transmitted infections in Black/African-American adolescents
Source: PLoS One. 2020 Nov 2;15(11):e0239650. doi: 10.1371/journal.pone.0239650 (PMC7605636; doi:10.1371/journal.pone.0239650)
Supplement: S1 File — (DOC) [file pone.0239650.s002.doc]

**The IMARA Program: Healthy Living for African American Women and their Daughters**

**Principal Investigator: Geri Donenberg, PhD**

**Institute for Juvenile Research (IJR)**

**Community Outreach Intervention Project (COIP)**

**Co-Investigators:**

**Erin Emerson, MA**

**IJR**

**COIP**

**Sally Freels, PhD**

**Epidemiology and Biostatistics**

**School of Public Health**

**Sponsor: National Institutes of Health**

**National Institute on Minority Health and Health Disparities (NIMHD)**

Background and Overview of the Study

Disproportionate rates of mental illness^6, 7^ and HIV/STIs among African Americans (AA) reflect significant health disparities.^14-18^ AAs account for more HIV/AIDS cases, people living with HIV/AIDS, and HIV-related deaths than any other racial group in the US.^1, 2^ In 2004, HIV was the 3^rd^ leading cause of death among Black women, and in 2006, AA women accounted for 66% of new AIDS cases among women.^3-5^ AA youth ages 13 – 19 comprise approximately 16% of US teens but 69% of new AIDS cases.^3^ Most infections among AA women and girls occur through sexual activity, and AA girls report more risky sex, less condom use, and lower perceived HIV risk than AA boys.^19, 20^ ^21^ Racial disparities also exist for AAs in rates of gonorrhea, chlamydia, and syphillis.^22^ Among 15 – 19 year old AA girls, gonorrhea rates are higher than any other race/age/gender group,^22^ and almost half of AA women have an STI.^23^ Important linkages exist between HIV/STIs and mental health;^10-12, 24-26^ Mental illness is linked to HIV through greater risk taking,^10-12, 24-26^ poor health promotion, and reduced effects of behavioral interventions^27-29^ ^11, 30^ for teens and adults.

HIV-risk factors extend beyond individual women or girls, yet few family-based, gender-specific, Afrocentric programs simultaneously address AA women, AA girls, mental health, and the mother-daughter dyad, thereby missing a critical opportunity to address HIV in a broader social context. Interventions that are sensitive to gender and culture focus on women (SISTA)^31^ or girls (SiHLE)^31^ and lack a mental health and family component. Family-based HIV prevention programs rarely address gender and culture or the adult family member’s HIV risks (Project STYLE). Simultaneously targeting multiple levels in an integrated program -- the mother-daughter dyad, women, and girls -- capitalizes on the reciprocal impact of mothers and daughters, and facilitates mutual reinforcement of prevention attitudes and behavior, thereby reducing intervention decay and sustaining positive outcomes over time.

IMARA (Informed, Motivated, Aware, and Responsible about AIDS) blends gender and ethnic components of SISTA and SiHLE (gender roles, ethnic pride, relationship power) with family and mental health components from Project STYLE (affect management, parental monitoring, adolescent development, parent-child communication) to create a culturally relevant, multi-level, integrated, family-based, HIV and mental health prevention program that simultaneously targets AA women and their daughters. Based on the Theory of Gender and Power,^32^ the Social-Personal model of HIV-risk,^10^ and findings from our research, IMARA emphasizes the interplay of family, peer, partner, and individual mechanisms as mediators of sexual risk taking for women and girls. Pilot testing (N=22 dyads) revealed strong feasibility, acceptability, and tolerability: >95% consent/assent rates, 96% retention at 2-month follow-up, and very positive feedback. Promising outcome data for mothers and daughters in targeted mediators (e.g., positive attitudes about HIV/AIDS, greater intentions to use condoms, increased parental monitoring, more open mother-daughter communication, more relationship power) and sexual risk outcomes (e.g., increased condom use, fewer partners) justify a randomized controlled trial.

This study has three specific aims:

(1) To conduct a 2-arm randomized controlled trial comparing IMARA to a family-based health program (FUEL^TM^). We will:

a. Randomly assign 300 14-18 year-old AA or black girls and their primary female caregivers to IMARA (N=150) or FUEL^TM^ (N=150). Women and girls will be recruited four ways: 1) from mental health clinics using clinic liaisons, 2) flyers will be posted in clinic recruitment sites and other agencies instructing interested families to call our recruiter, 3) IMARA participants will hand flyers to interested women and girls they know, and 4) COIP field station staff will pass out flyers and recruit interested women and girls at the field stations and in the community. We will examine the effects of IMARA on women and girls’ sexual behavior at 6- and 12-months.

(2) To evaluate the impact of IMARA on theoretical mediators posited by the Theory of Gender and Power and the Social-Personal framework associated with AA women and girls’ risky sex. We will:

a. Assess changes in women and girls’ *Individual Attributes* (HIV/AIDS knowledge, attitudes, and beliefs, mental health/emotion regulation, ethnic identity); *Peer and Partner Processes* (partner characteristics, relationship power dynamics, peer influences, partner communication); and *Family Context* (mother-daughter relationship and communication, parental monitoring) at baseline and follow-ups.

b. Evaluate mediation and moderation of theoretical mechanisms on women and girls’ sexual behavior.

1. To assess the impact of IMARA compared to FUEL^TM^ on sexually transmitted infections (STIs). We will:

a. Test women and girls’ urine for three common STIs at baseline and 12-month follow up.

b. Explore linkages between biological outcomes and targeted mediators and moderators of change.

Hypotheses

IMARA participants will report less risky sex (fewer partners, more consistent condom use, and later sexual debut among non-sexually active girls) at 6- and 12-months and have fewer incident STI infections at 12-months; and (b) IMARA participants will report positive changes in theoretical mediators: individual attributes (more positive condom attitudes, self-efficacy, ethnic pride), peer/partner influences (more relationship power, partner communication, and awareness of partner influences on sexual decision making), mental health (improved emotion regulation and understanding of the links between mental illness and risky sex), mother-daughter communication (more open and comfortable), and mother-daughter relationships (more parental monitoring and warmth, less parental permissiveness).

Significance

This proposal answers a compelling need for innovative prevention programs that address the intersecting health disparities of mental illness and HIV-risk among AA or black women and girls. IMARA simultaneously targets two high-risk groups, AA or black women and their daughters. IMARA capitalizes on a key incentive for women to reduce their risk, as evidence documents the reciprocal impact of parents and children, and IMARA facilitates mutual reinforcement of safer sex behavior. The effects of individual-level programs decay over time for adults and youth.^33, 34^ Parent involvement may enhance long-term outcomes for youth, because mothers can continue prevention messages after the formal program ends and tailor them to their daughters’ developmental level. The desire to be a positive role model for their daughters may also reduce decay for women. IMARA is uniquely responsive to AA cultural values that emphasize strong families, extended kinships, and close interpersonal relationships. The process of teaching daughters to engage in healthy behavior may be an important catalyst stimulating change in women’s sexual behavior.

Methods

Overview of Procedures

In months 1 – 6, we will hire and train staff, refine measures, prepare data collection instruments, re-establish recruitment and retention procedures, obtain all necessary ethics approvals, prepare intervention materials and treatment fidelity procedures, train facilitators, and finalize operations manuals. We will replace instruments that have more current evidence of reliability and validity. We will finalize data entry methods and program ACASI for data collection. All staff will document their human subjects training and sign confidentiality agreements. We will obtain a certificate of confidentiality and re-convene GIRLTALK’s CAB to review the research protocol, recruitment and data collection strategies, and intervention delivery plans. We will prepare all intervention materials and establish random assignment procedures. We will train assessors and facilitators and re-establish relationships with mental health clinics and site liaisons to prepare for subject recruitment. In months 7 – 57, we will use the recruitment, assessment, tracking, and intervention procedures successfully established in GIRLTALK and the pilot study to enroll approximately 300 women and their 14-18 year-old daughters. Assuming 15% attrition from baseline assessment to the intervention (see Project STYLE), we expect to assess 355 dyads in order to randomly assign 150 dyads to each condition. Follow up assessments will occur at 6- and 12-months, and we will test women and girls for Chlamydia, Gonorrhea, and Trichomonas at baseline and 12-months. A UIC adolescent medicine physician, or one of her associates, will treat all participants who test positive for an STI. The UIC adolescent medicine physician or her associates will have access to UIC clinic records of those subjects who choose to be treated by her. Participants will be asked to provide a code word when they submit their urine sample for testing. This code word will be unique to them and something they will remember. This code word will be described to the participants as being: a word that only they will know and that other family members, including their mother/daughter, will not know, a word that the participant will be sure to remember, and a word that they will not share with anybody else. If participants ask for guidance in choosing a code word, we will suggest that participants might use the name of their favorite actor, musician, or favorite book. We will stress that participants are not to share their code word with their mother/daughter and the word should not be a word that is easily identifiable by anyone else. This code word, in addition to participants’ full name and date of birth, will then be used to verify identity and as an extra precaution to maintain confidentiality when disclosing STI test results.

We will re-assemble the GIRLTALK (Protocol # 2002-0820) CAB to consult, evaluate, and guide our procedures, materials, and measures. The CAB includes local stakeholders, service providers, and AA women and daughters in psychiatric care and will strengthen the project’s linkages and relevance to the community. The CAB will meet annually to ensure that all activities are conducted with the highest standard of excellence and scientific integrity.

We will use the recruitment, assessment, tracking, and intervention procedures successfully established in GIRLTALK and the IMARA pilot study (Protocol # 2002-0820 "Family Predictors of HIV-Risk in Mentally Ill Gils") to enroll approximately 300 women and their 14-18 year-old daughters. Women and girls will be recruited four ways: 1) from mental health clinics using clinic liaisons, 2) flyers will be posted in clinic recruitment sites and other agencies instructing interested families to call our recruiter, 3) IMARA participants will hand flyers to interested women and girls they know, and 4) COIP field station staff will pass out flyers and recruit interested women and girls at the field stations and in the community. Assuming 15% attrition from baseline assessment to the intervention, we will assess 355 dyads in order to randomly assign 150 dyads to each condition. Follow up assessments will occur at 6- and 12-months, and we will test (via urine screen) women and girls for Chlamydia, Gonorrhea, and Trichomonas at baseline and 12-months. A UIC physician, or one of her associates, will treat all participants who test positive for an STI. The UIC adolescent medicine physician or her associates will have access to UIC clinic records of those subjects who choose to be treated by her.

Consistent with our prior recruitment success, we will: (1) post flyers and brochures at clinic recruitment sites and other agencies such as COIP field stations, community centers, churches, and schools interested in IMARA, inviting families to call us if they are interested; (2) hire a clinic staff member at each clinic recruitment site and COIP field staff at COIP field stations, to identify eligible participants, inform families of the project, and request mothers’ permission to be contacted by our research team; (3) accompany clinic recruitment site recruiters and COIP field staff recruiters to community outreach efforts; (4) place IMARA recruitment staff at recruitment sites to provide brochures and information about the study to potential participants; (5) mail interested families a letter with details about the study; (6) telephone interested families to explain the study, including random assignment, and ask permission to do a home visit in order to explain the study face-to-face; (7) schedule the baseline interview and workshop dates; (8) mail confirmation letters with appointment dates and times; (9) telephone families the day before each appointment/session to confirm attendance; (10) provide transportation to the site as needed, and (11) at the end of workshop intervention day 2 encourage participants to tell other families they know about IMARA and pass along flyers and brochures to interested families.

Women and daughters will complete assessments at baseline, 6-, and 12-months. At baseline, we will review the consent/assent forms separately with women and girls. Trained staff will administer questionnaires and interview women and girls separately to ensure confidentiality. Dr. Donenberg is a licensed clinical psychologist and will provide clinical backup in cases of suspected child abuse, neglect, and suicide. Consent/ assent forms will state the exceptions to confidentiality, and where a girl or mother reports child abuse or neglect or suicidal ideation or attempts, a clinician will be consulted. We have used this procedure effectively for 12 years to ensure the safety of our participants. Mothers and girls will each receive $30 to complete the baseline assessment. Mothers will receive an additional $10 for travel and parking. If daughters come in without their mothers to complete an interview, then we will pay the daughter $10 for travel and parking. A month before follow-ups, we will contact families to request their participation in the next wave of data collection. To increase participation for families who cannot come to the clinic, we will provide transportation or conduct interviews at a convenient location (e.g., library, park district) as needed. For those subjects who were consented prior to June 3, 2013 (when the compensation amounts changed in Amendment 9), we will pay the overall difference in compensation amounts, which is $30 for mothers and $20 for daughters, at the 12-month assessment. This ensures that all participants receive the same amount of overall compensation.

We will hire a retention coordinator to track subjects and address scheduling issues and barriers. She will telephone, text message, and/or e-mail families as appointment reminders. We will collect phone numbers, e-mail, and home addresses of at least three people who could help us locate them in the future. Based on prior experience, we will ask for contacts living in stable housing and family pastors. We will telephone, e-mail, and/or text families monthly to update our lists, and we will verify participants’ locating information each time we contact them. We will establish a toll-free number and project e-mail address. We will mail handwritten thank you cards after each interview/workshop to demonstrate our appreciation. We will send personalized birthday cards and give small gifts to sustain interest in the study. Two months after baseline, we will mail families a newsletter with fun facts about AA history and a list of activities or events around Chicago. We will include postage-paid address change cards with our toll-free number and e-mail address. If our correspondence is returned, we will telephone, e-mail, or text message families to obtain their new address. If we do not reach the family after one week and at least five attempts, we will contact people from participants’ records and use well-known internet locator services. We will send trackers to the family’s last known address and leave letters at homes asking families to call us. We will return to residences to ensure letters were received. Prior experience has taught us to ring all doorbells, visit homes after school, and wait for someone to return. If the family no longer lives at the address, we will ask the family’s contacts to help us locate them. At baseline, we will request permission to obtain forwarding information from girls’ schools.

We will use a 1:1 randomization allocation to assign 150 mother-daughter dyads to IMARA or FUEL™ on day one of the intervention. We will enroll 12-16 families per cohort (i.e., 6-8 into IMARA and 6-8 into FUEL™), and we will enroll approximately one cohort per month.

We will deliver IMARA and FUEL™ in groups of 6-8 on two consecutive Saturdays. Two AA females will co-lead each intervention. We will provide breakfast and lunch. All research activity will occur at IJR, COIP (UIC’s School of Public Health and field stations), or locations convenient for families (their homes, community centers, libraries, churches, etc.).

IMARA: IMARA blends three programs with the most relevance for AA women (SISTA) and girls (SiHLE) and families in psychiatric care (Project STYLE). Separate mother and daughter groups cover parallel content and run simultaneously, and joint activities enhance mothers’ credibility as a resource for HIV/STI prevention, practice new communication skills, negotiate conflict, and strengthen the mother-daughter relationship. Activities reinforce the reciprocal impact of mothers and daughters, and enhance safe sex knowledge, attitudes, and skills. Morning and afternoon sessions begin with an icebreaker and/or poem to enhance ethnic and gender pride. IMARA’s goals and motto are presented to emphasize strong mother-daughter relationships, foster sisterhood, build group cohesion, and increase motivation. Ground rules are reviewed, and each woman and girl signs the IMARA pact to confirm her commitment to the program. At the end of day 1, mothers and daughters receive homework for the week. Woven throughout IMARA is the impact of alcohol and drug use on risk behavior, including condom use while high. IMARA’s curriculum is provided in this submission (see Appendix 1).

FUEL™ Health Promotion Control Group: mothers and daughters randomly assigned to FUEL™ will participate in separate 2-day workshops identical in length and intensity to IMARA. FUEL Health Promotion Control combines two programs, FUEL and Project Balance Health Promotion. FUEL™ promotes healthy activities by encouraging good nutrition, exercise, and informed consumer behavior. FUEL™ does not explicitly address HIV/STI prevention. However, the high prevalence of HIV/STIs among AA women and girls make it ethically questionable to withhold all information about HIV/STIs. Thus, we will present information from Balance’s session about HIV/AIDS, condoms, and other STIs. We will also insert the following sessions from Balance into FUEL: alcohol use, drug/marijuana use, nutrition, exercise, and violence to increase program length. The FUEL™ curriculum is provided in this submission (see Appendix 2).

Eligibility Criteria

Girls will be included in the study if they: a) self-identify as Black or AA; and b) 14-18 years-old; Women will be included in the study if they: a) self-identify as Black or AA primary female caregivers who live with the girl, and b) over 18 years old. Women and daughters must agree to participate as a dyad, and girls’ refusal will supersede parental consent.

Girls and women will be excluded from the study if they: a) are unable to understand the consent/assent process; b) do not speak English, because instruments are normed for English speakers; c) are actively psychotic or severely mentally ill; and d) girls do not live with the participating primary female caregiver**.**

Grand Total UIC Participants: 355 mother/daughter dyads (total 710)

Justification for inclusion of any special or vulnerable populations

AA girls in psychiatric care are at disproportionate risk of HIV/AIDS/STIs. We are targeting 14–18 year old girls because: (1) One aim of the intervention is to delay sexual debut. Few AA girls initiate sex before age 13 but by 12th grade, 66.9% of AA girls have had sex. In GIRLTALK (the basic longitudinal study from which the current study is based), the average age of debut was 14 years. Hence, 14 – 18 year olds are likely to be on the cusp of initiation and can benefit from the program. (2) Components of the intervention were tested with 14-18 year olds with no adverse effects and no age differences in treatment outcomes. (3) 14 –18 year olds are often grouped in HIV prevention studies and are in school together. They are often in the same peer group and exposed to each other’s’ activities, even if they differ in their level of sexual experience. Thus, they are usually aware of and exposed to the variety of activities across this age range, thereby minimizing developmental differences. (4) We have extensive experience with this age range.

Recruitment Procedures

Participants will be recruited four ways:

1) A clinic staff member from each clinic recruitment site (not our research staff) will review their own site records and identify eligible families, inform the family of the project and confirm eligibility requirements using the Eligibility Screening Questionnaire for Clinic Recruitment Sites or Field Recruitment.

The community clinic liaison will document teens’ gender, age, and ethnicity. She/he will also document the primary female caregiver’s age and confirm she is living with the teen. If both the teen and parent meet eligibility requirements she/he will inform them of the project and request permission to release their contact information to study recruiter. The study recruiter will confirm eligibility requirements using the Eligibility Screening Questionnaire for Clinic Recruitment Sites or Field Recruitment.

UIC study staff will not have access to or review any clinic records and no research staff will contact families without explicit permission by the caregiver.

Clinic staff will not be screening types of mental health issues or diagnoses; however if it is known via records that the adolescent is severely mentally ill and/or psychotic she will not be eligible and the female caregiver will not be contacted.

Study recruiters will also accompany clinic recruiters to community outreach efforts and be at recruitment sites to provide brochures and information about the study to potential participants.

2) IMARA Program Flyers and IMARA Brochures will be posted at clinic recruitment sites and other agencies such as COIP field stations, community centers, churches, and schools interested in IMARA, inviting families to call study staff if they are interested. If interested families call study staff, a study recruiter will use the Eligibility Screening Questionnaire for Flyer/Brochure to assess eligibility requirements.

3) IMARA participants will hand IMARA Program Flyers to interested women and girls they know. At the end of workshop intervention day 2 facilitators will encourage participants to tell other families they know about IMARA and pass along IMARA Program Flyers and IMARA Brochures to interested families. If interested families call study staff, a study recruiter will use the Eligibility Screening Questionnaire for Flyer/Brochure to assess eligibility requirements.

4) UIC COIP field station staff will pass out IMARA Program Flyers and IMARA Brochures and recruit interested women and girls at the field stations and in the community. COIP field staff at COIP field stations and other community locations will identify eligible participants, inform families of the project, and request mothers’ permission to be contacted by our research team. Study recruiters will also accompany COIP field recruiters to community outreach efforts.

COIP field station staff will use the Eligibility Screening Questionnaire for Field Recruiters to confirm eligibility requirements and document teens’ gender, age, and ethnicity. She/he will also document the primary female caregiver’s age and confirm she is living with the teen. If both the teen and parent meet eligibility requirements she/he will inform them of the project and request permission to release their contact information to study recruiter. The study recruiter will then contact the family and confirm eligibility requirements using the Eligibility Screening Questionnaire for Clinic Recruitment Sites or Field Recruitment.

For families who respond to the IMARA Program Flyer and/or IMARA Brochure, the study recruitment coordinator will screen for eligibility using the Eligibility Screening Questionnaire for Flyer/Brochure.

Families who agree to be contacted by study staff will receive written information about the study and a telephone call from the study’s recruiter. Eligibility criteria will be reiterated in the letter of information, and the study’s recruiter will further assess eligibility when speaking with families directly. The study recruiter will ask permission to visit with the mother at home in order to explain the study face-to-face and establish a relationship.

Contact information of families who do not wish to take part in the study will not be retained for research purposes.

If there are questions about the teen’s cognitive or mental abilities, the study recruiter will consult with the study’s Project Coordinator and/or Principal Investigator.

Note that during the consent process there is an additional screening to determine if participants understand the consent/assent process as outlined in Appendix B. If the participant cannot answer questions about the study (e.g., name the things they will be expected to do during the study, explain what they would do if they no longer wished to participate, what are the risks for participating, etc.), the study staff will consult the Project Coordinator or Principal Investigator to determine whether the participant is eligible.

For participants requiring transportation for an interview, STI treatment, or other research-related activity, we will offer to provide auto transportation from their home or other safe location (such as a school) to research activities and back. This service will be optional and participants are given other transportation options including but not limited to CTA or the participant’s personal vehicle.  All drivers on the project are staff members who have a valid driver’s license. A second staff member will be present in the car at all times if minors under the age of 18 are being transported.  All procedures related to transportation of research subjects have been reviewed by the risk management department of University of Illinois to ensure any relevant liability issues have been considered.

For research assessments, we will transport only one dyad at a time. Unless both individuals request transportation together, for STI treatment we will only transport one individual (mother or daughter) at a time to preserve mothers and daughter's confidential test results. For dyads that receive transportation to baseline assessments, we will ask them their transportation preferences for the workshop sessions; we will give mothers and daughters each the option of being picked up with other mother-daughter dyads in the same car since they will be participating in the intervention groups together and learn of each other’s involvement. If either member of the dyad says no, we will ask if they would be willing to be in a car with other participants if we picked them up from an alternate location that is not their home. If they say no, we will make alternate arrangements for their transportation, such as arranging and paying for taxi service.

Assessment Procedures and Measures Used

Assessments will take place at UIC’s Community Outreach Intervention Projects (COIP), Institute for Juvenile Research, COIP field stations, participant’s home, or other convenient locations for families.

Measurements. We have used most of the measures with AA women and girls in psychiatric care (see Appendix B), but we will review them in months 1 - 3 for semantic and content relevance. We will administer most measures via voice-activated computer (ACASI), but interviewers will remain in the room to ensure comprehension. ACASI offers several advantages: (1) It yields more accurate responses than face-to-face interviews or written questionnaires about sensitive topics.283-285 (2) Subjects can complete measures at their own pace. (3) Questions are answered privately increasing anonymity. (4) The interview structure permits skipping follow-up questions when the stem is answered negatively. (5) Computer delivery ensures uniform administration and bypasses reading problems. (6) ACASI has been used successfully across many studies of parents and youth in psychiatric care.12

We have used most of the measures with AA women and girls in psychiatric care (see Appendix 3), but we will review them in months 1 - 3 for semantic and content relevance. We will administer most measures via voice-activated computer (ACASI), but interviewers will remain in the room to ensure comprehension.

We will evaluate the intervention using observer feedback, participant evaluations, and facilitator feedback.

Demographics. We will ask if participants heard about IMARA from someone who has already participated in the program. We will collect residential and e-mail addresses, home and cell phone numbers, and contact information for at least 3 people who can help us locate families in the future. We will collect maternal age, ethnicity, education, and income, girls’ age, family structure, neighborhood quality, psychiatric history, and the role/presence of male caregivers in the home. We will explore the role of these factors on treatment outcomes and mediators. A waiver of consent to collect information on the adolescent’s siblings was granted by UIC’s IRB.

Individual Attributes. HIV/AIDS–related cognitions and skills: HIV/AIDS/STI Knowledge, completed by mothers and daughters is a true-false survey that measures transmission routes, misconceptions about transmission, and risk-reduction strategies.286-288 Women and girls will indicate their general attitudes about HIV/AIDS, peer norms regarding HIV/AIDS prevention, intentions to prevent HIV/AIDS, attitudes toward HIV/AIDS preventive acts/condom use289 and their HIV/AIDS behavioral skills (self-efficacy to prevent transmission, apply condoms, and negotiate with a partner).287, 289 Women will report on their beliefs about concurrent partnerships with The Relationship Issues Scale,290 a measure of attitudes and expectations regarding relationship exclusivity and non-exclusivity. Girls will also complete items from the sexual non-exclusivity scale from The Relationship Issues Scale290.

Mental health and emotion regulation: The Child Behavior Checklist (CBCL)291 and Youth Self-Report (YSR) are widely-used and validated, parent and youth measures of child behavior problems. Both generate raw and T-scores for internalizing (e.g., sadness, anxiety) and externalizing (e.g., fighting, swearing) syndromes.291,292 Women and girls will complete The Toronto Alexithymia Scale (TAS), a measure of emotion regulation, externally oriented thinking, and the ability to identify and describe feelings.293 Women will indicate their own mental health on the Symptom Checklist-90-R (SCL-90R).294

Ethnic pride: Women will complete the private regard scale of The Multidimensional Inventory of Black Identity (MIBI),295 and girls will complete the Pro-Black scale of the Adolescent Survey of Black Life.296

Peer and Partner Influences. Partner relationship characteristics and power dynamics: Women and girls will report on their partners’ age, perceptions of control over partners’ condom use, partners’ resistance to condom use, and financial dependence on partners.53 Women and girls will complete measures of relationship characteristics: (1) Power and Attitudes in Relationships (PAIR) scale assesses gender roles and norms, perceived need to be in a relationship, women and men’s division of household responsibility, and sexual assertion and power in decision-making.245 (2) The Sexual Relationship Power Scale (SRPS) measures perceived relationship control and decision-making dominance. Partner sexual communication: Women and girls will indicate whether they ever talked to their partner about a list of sexual topics, how often they were discussed, whether the conversation was open, and how comfortable they felt. Items were adapted from the Sexual Risk Behavior Questionnaire297 and Miller et al. (1998).174

Interviewers will also administer the Adolescent Romantic Relationships Scale. Items were adapted from the National Longitudinal Study of Adolescent Health (ADD Health Study) 346 characterizing romantic relationships. Items assess a continuum of dating and sexual activities (e.g., went out together in a group, held hands, kissed, had sexual intercourse).

Peer Norms: Girls will report on peer approval of sex, alcohol, marijuana, and cigarette use. Items combine two scales on the Health Questionnaire,298 a widely-used measure of adolescent health behavior with extensive reliability and validity.299-301 Girls will also indicate their association with peers who get good grades and participate in extracurricular activities and community groups.298

Family Context. Affective characteristics: Girls and mothers will indicate the strength of their attachment using the Inventory of Parent and Peer Attachment (IPPA).302, 303 Instrumental characteristics: Mothers and girls will complete the Parenting Style Questionnaire (PSQ)304 measuring parental supervision, monitoring, and permissiveness. Sexual communication: We will assess girls’ and mothers’ perceived quality and quantity of risk-specific communication on several topics (e.g., having sex, condoms, and AIDS).174 187, 209

Outcome Variables. Risky Sexual Behavior. The AIDS-Risk Behavior Assessment (ARBA)12 is a computer-assisted interview of sexual behavior and drug use. The ARBA was derived from five established measures used in large-scale studies to examine HIV/AIDS-risk in youth and adults. 305,306,307-309,310 The ARBA assesses substance use, sexual behavior, and needle use. We will assess mothers and daughters’ condom use, sex with high-risk partners, sex while using drugs/alcohol, number of partners, sexual debut -- and relative frequency and count-based indicators.

We will measure women’s and girls’ STIs using biological endpoints (yes/no) to evaluate intervention effects. We will screen urine for three sexually transmitted pathogens at baseline and 12- month follow up; N. gonorrhoeae, C. trachomatis, and T.vaginalis. We will use nucleic acid amplification technologies (NAAT) for STI testing.311, 312 Consistent with our other studies, we will provide women and girls with collection containers and escort them to a private, secure room in which to produce the specimen. Participants will be asked to provide a code word when they submit their urine sample for testing. This code word will be unique to them and something they will remember. This code word will be described to the participants as being: a word that only they will know and that other family members, including their mother/daughter, will not know, a word that the participant will be sure to remember, and a word that they will not share with anybody else. If participants ask for guidance in choosing a code word, we will suggest that participants might use the name of their favorite actor, musician, or favorite book. We will stress that participants are not to share their code word with their mother/daughter and the word should not be a word that is easily identifiable by anyone else. This code word, in addition to participants’ full name and date of birth, will then be used to verify identity and as an extra precaution to maintain confidentiality when disclosing STI test results.

Staff will decant urine specimens to centrifuge tubes labeled with unique subject-sample identifiers and store them in refrigerators. We will maintain subject-sample ID linkages in confidential logs kept in secure, locked storages. Upon processing the specimens, the laboratory will email the coded results to us within one week. We will offer women and girls who test positive for an STI pathogen (chlamydia, gonorrhea, or trichomonas) the most current and effective treatment available. A UIC adolescent medicine physician or her associate will oversee STI treatment. The UIC adolescent medicine physician or her associate will have access to UIC clinic records of those subjects who choose to be treated by her. We have been following these procedures for nine years without any adverse events.

After collecting the urine sample we will send the specimen to Emory University, Caliendo Laboratory in Atlanta, Georgia. We will inform women and girls that the urine cup will be labeled with unique code numbers and the lab request form will contain the code number and no other identifying information. This is the procedure for Caliendo Laboratory and it is done in effort to reduce risks of potential breaches of confidentiality. The lab will report the STI testing results to us and we will inform the participant of their test results.

The data manager will maintain a master database with each participant’s name, research number, and unique STI testing code number. She will enter in each participant’s name and the database will self-populate with a research number, providing a link between the participant’s identity and her research number. STI testing numbers will consist of the participant’s research number with either the letter ‘Y’ or ‘M’ after the number, the letter ‘Y’ will be used for adolescents, and ‘M’ for female caregivers. Links between participants’ identities,research numbers, STI testing numbers, and STI test results will be verified by at least two staff members (the project director, data manager, and/or recruitment coordinator) prior to reporting any STI results to participants.

The following procedures will be followed: (1) STI testing numbers will be linked to research participant numbers in the master database prior to beginning baseline assessments, (2) STI testing numbers, names, and research numbers will be stored in this master database, (3) after completion of the baseline assessment, links between STI testing numbers, names, and research numbers will be verified by at least two staff members (the project director, data manager, and/or recruitment coordinator) using the Master List Verification Log and these two staff members will sign and date the Master List Verification Log. This Master List Verification Log will be kept in a locked cabinet in the Project Director’s office. Only the data manager, recruitment coordinator, and Project Director will have access to this log. (4) The link between each STI result and research number/participant name will be provided to the data manager by the project director on an STI verification log, to be verified within 5 days of entry (before any STI results are reported), (5) at least two staff members (the project director, data manager, and/or recruitment coordinator) will each sign the STI verification log recording the date that STI result verification occurred. This STI Result Verification Log will be kept in a locked cabinet in the Project Director’s office. Only the data manager, recruitment coordinator, and Project Director will have access to this log. (6) Prior to reporting STI results, the data manager and project director will confirm that data verification was completed.

If the participant tests positive and chooses to get treated by a UIC adolescent medicine physician, our study consultant, we will forward the results to the UIC adolescent medicine physician or her associate in the Adolescent Medicine Clinic at UIC, who will provide the participant with the appropriate treatment, and create a medical record. The UIC adolescent medicine physician or her associate will have access to UIC clinic records of those subjects who choose to be treated by her. Participants will be asked to provide a code word when they submit their urine sample for testing. This code word will be unique to them and something they will remember. This code word will be described to the participants as being: a word that only they will know and that other family members, including their mother/daughter, will not know, a word that the participant will be sure to remember, and a word that they will not share with anybody else. If participants ask for guidance in choosing a code word, we will suggest that participants might use the name of their favorite actor, musician, or favorite book. We will stress that participants are not to share their code word with their mother/daughter and the word should not be a word that is easily identifiable by anyone else. This code word, in addition to participants’ full name and date of birth, will then be used to verify identity and as an extra precaution to maintain confidentiality when disclosing STI test results.

We are required to disclose positive STI results to the participant's local Department of Health. The report includes what STI the participant was diagnosed with, the date the positive test was collected and the participant's name, address, phone number, date of birth, age, sex, race/ethnicity, and the treatment they received. The UIC adolescent medicine physician or her associate will inform us regarding whether the participant received treatment.

We disclose the above procedures in the assent document and in the consent forms we inserted HIPAA authorization language to further clarify what information we may be disclosing to the Department of Health. This disclosure is compelled by state law and not a breach of confidentiality.

Other measures. We will evaluate additional areas related to HIV-risk behavior. Mothers will report their trauma experiences using the Trauma History Questionnaire. Girls will report PTSD symptoms using the UCLA PTSD Index. Girls will also report their history of sexual abuse.93 We will also assess girls’ and women’s religious perceptions and involvement adapted from ADD Health. Participants will also complete a measure assessing “cross-talk” and report whether they talked to other participants in the other program and what they talked about.

Girls and mothers will complete 5 items measuring sexual self-agency. 349

Girls and mothers will complete the Stress and Coping with Racism Microaggressions measure. This questionnaire assesses experiences of racial microaggression, the perceived stress of these experiences, and secondary control coping in response to these experiences. The first part includes 12 items that ask about participants' experiences with racial microaggressions, and the items are based on the Racial and Ethnic Microaggression Scale 347 (REMS). The second part includes 11 items that ask about participants' coping in response to these experiences, and the items are based on the secondary control coping subscale of the Response to Stress Questionnaire 348.

Mothers and girls will each receive $30 to complete the baseline assessment. Mothers and girls will each receive $25 to complete the 6-month follow up assessment and $30 for the 12-month follow up assessment. Mothers will receive an additional $10 for travel and parking after each interview. If daughters come without their mothers to complete an interview, we will pay daughters the $10 for travel and parking. Participants will be compensated upon completion of the baseline, 6 and 12 month assessment. Partial payments will not be made. If either or both mothers and daughters test STI-positive, treatment will be provided for free. For those subjects who were consented prior to June 3, 2013 (when the compensation amounts changed in Amendment 9), we will pay the overall difference in compensation amounts, which is $30 for mothers and $20 for daughters, at the 12-month assessment. This ensures that all participants receive the same amount of overall compensation.

Intervention Delivery

We will ensure treatment fidelity per the Treatment Fidelity Workgroup of the NIH Behavior Change Consortium:269 (1) Clear, detailed manuals and facilitator guides; (2) Standardized training protocols; (3) Training to competency; (4) Session observations; and (5) Frequent communication between facilitators, investigators, observers, and the CAB. Facilitators will complete measures of treatment adherence at the end of each day and participate in debriefing sessions and supervision. An observer will rate each session to verify that the intervention was delivered as planned.

We will use a 1:1 randomization allocation to assign 150 mother-daughter dyads to IMARA or FUEL™ on day one of the intervention. Randomizing on day one worked well in Project STYLE to eliminate attendance bias; treatment arms were equivalent at baseline. We will enroll 12-16 families per cohort (i.e., 6-8 into IMARA and 6-8 into FUEL™), and we will enroll approximately one cohort per month.

We will deliver IMARA and FUEL™ in groups of 6-8 on two consecutive Saturdays. Two AA females will co-lead each intervention. We will provide breakfast and lunch. At the end of the intervention workshop day one, mothers and daughters will each receive $40 after completing a day one evaluation form. Mothers will be paid an additional $10 for transportation costs. At the end of the intervention workshop day two, mothers and daughters will each receive $40 after completing a day two evaluation form. Mothers will be paid an additional $10 for transportation costs. For those subjects who were consented prior to June 3, 2013 (when the compensation amounts changed in Amendment 9), we will pay the overall difference in compensation amounts, which is $30 for mothers and $20 for daughters, at the 12-month assessment. This ensures that all participants receive the same amount of overall compensation. A 2-day workshop has several advantages over multi-session interventions: (1) It maximizes participation so families can benefit from the full program. In Project STYLE and IMARA’s pilot study, families requested 1 – 2 full-day meetings, because the burden to attend weekly sessions is high, places undue strain on their financial resources, and interferes with children’s activities. (2) Separating the two sessions by a week has the added benefit that women and girls can practice new skills, integrate content, problem-solve barriers to success, and receive feedback about barriers to change. (3) Families in outpatient psychiatric care often suffer stressors that hamper their ability to attend a multi-session intervention. Two sessions have real-life utility; they are pragmatic and responsive to the needs of urban low-income families and increase the likelihood of dissemination. (4) Research supports psychosocial270-272 and behavioral change among women and teens following brief HIV/AIDS prevention interventions.258, 272-280 For example, Jemmott et al. (2007)276 demonstrated increased condom use, less unprotected intercourse, and fewer STI diagnoses at 12-month follow-up among 18-45 year-old inner-city AA women who received two 40-minute group sessions. Jemmott et al. (2005)281 compared three 4-hour interventions for AA and Latina girls (M= 15.5 years) recruited from an adolescent medicine clinic. Girls in the skills-based condition reported less unprotected sex at 12-months, fewer partners, and were less likely to test STI positive. Similarly, in Project STYLE, teens in the family-arm reported more condom use and less risky sex than youth in the health promotion control condition at 6-month follow up. (5) The briefer format is more likely to be adopted by mental health and community agencies, with limited resources to deliver intensive weekly HIV-prevention programs.276 (6) IMARA’s pilot data revealed an overall pattern of change in targeted mediators and sexual outcomes for mothers and daughters (see Preliminary Studies).

IMARA overview and rationale. We evaluated existing HIV prevention programs for youth and adults, their underlying theories, feasibility, and empirical evidence. We found no family-based intervention that simultaneously targeted women and their daughters’ risk behavior from an Afrocentric perspective or the impact of mental health, family context, gender, power, and ethnic pride. We identified three programs with the most relevance for AA women (SISTA)31 and girls (SiHLE)253 and families in psychiatric care (Project STYLE) that addressed our hypothesized mediators and moderators (see Figure 1) of AA women’s and girls’ risky sexual behavior. We blended the unique strengths of each curriculum to create IMARA and piloted the curriculum with 22 mother-daughter dyads.

SISTA. SISTA (Sisters Informing Sisters about Topics on AIDS)31 is a 5-session, individual-focused, peer-led, skills-based intervention to prevent HIV infection in AA women. SISTA addresses cultural influences on sexual behavior, the sexual division of labor and power, and gender-specific standards for appropriate sexual conduct in heterosexual relationships.31 Findings from an RCT of 128 sexually active AA women revealed improved condom use, communication, and assertiveness.31 SISTA has been widely distributed,43, 256 with more than 700 US agencies trained to deliver it,255 and the largest network of community health centers implemented SISTA with AA women at high risk of HIV in Illinois.43

SiHLE. SiHLE (Sistering, Informing, Healing, Living, and Empowering),255 a 4-session, 16-hour, HIV prevention program for 14 – 18 year old AA girls, is similar in theoretical, methodological, and core elements to SISTA. SiHLE emphasizes ethnic and gender pride. Sessions address the impact of older male partners, abusive partners, stereotypes of AA teens, serial monogamy, peer pressure to have sex, and safer sex communication.255 SiHLE stresses abstinence, HIV-risk reduction strategies, healthy relationships, condom use, and self-efficacy, especially to refuse unsafe sex with partners.253 In an RCT, SiHLE participants reported more consistent condom use, greater likelihood of using condoms at last intercourse, less likelihood of new sex partners, and better condom application skills compared to controls.253

Project STYLE. Project STYLE is a family-based HIV prevention program for 13 – 18 year olds with severe mental illness282 based on the Social-Personal Framework of HIV-risk.13 Using cognitive-behavioral strategies to address family factors associated with HIV prevention, Project STYLE emphasizes parental modeling, effective communication and decision-making skills, improving parent-teen communication, and strengthening parental monitoring and supervision. Separate parent and youth groups cover parallel content and run simultaneously. Youth and parents join together to practice communication styles and new skills and receive group feedback. Parents and teens learn general HIV/STI information and assertive communication. Personal vulnerability is emphasized with a focus on teens with mental illness. Assertive communication skills are applied to sexual discussions and communication about HIV/STI risk behaviors with parents and partners, and teens discuss risk situations, behaviors, and solutions that include parents as partners in staying safe. Parents create an individualized monitoring plan for their teen. Parents and youth learn condom use skills, and practice applying condoms separately and together to portray parents as useful resources in sex education. The combined parent-teen portion applies the knowledge learned separately by having parents and teens role-play positive and negative communication. Dyads discuss the monitoring plan, receive feedback from group members about their communication, and re-do the conversation incorporating group feedback**.** Parents and teens discuss their values about youth sexual behavior, and conclude by creating a public service announcement to reflect what they learned.

**Table 1. IMARA Curriculum: Theoretical Constructs, Activities and Target Participant**

| **Theoretical Constructs** | **IMARA Activities** | **Tx^1^** | **Target^2^** |
| --- | --- | --- | --- |
| Individual Attributes |  |  |  |
| Ethnic and gender pride | Poems: A Room Full of Sisters, Phenomenal Woman  Music, movies, TV images that portray AA women (positively, negatively)  Successful AA women game: Name that Woman!  Young, Black, and female: Listing positive characteristics, stereotypes  AA women role models and AA women who shaped our history | Sa, Si  Si  Si  Si  Si | J  W,G  J  W,G  W,G |
| HIV/AIDS/STI cognitions and skills  Knowledge, Attitudes, Beliefs  Personalizing risk  Behavioral skills | HIV/AIDS jeopardy; Virus carrier handshake; High, moderate, low risk situations  Videos Bloodlines and Out of Control: AIDS in Black America; Discussion  Identifying risk triggers (people, places, feelings); Developing individual risk plans  LIPSTICK (acronym for condom use steps) | St  St  St  St | W,G  W,G  W,G  W,G,J |
| Mental health/Emotion regulation  Overcoming challenges | Feelings as triggers for risk behavior; Feeling thermometer; Healthy coping  Links between mental health and risk behavior  Observed mother-daughter conflict discussion  What matters most – Distinguishing values; Taking care of you; Value of my body  Poem: Still I Rise (Maya Angelou)  Phenomenal Woman (Maya Angelou) scavenger hunt; Graduation ceremony | St  St  St  Si  Si,Sa  Sa,Si | W,G  W,G  J  W,G  W,G  J |
| Family Context |  |  |  |
| Instrumental characteristics  Parental monitoring | Creating personalized monitoring plans | St | W |
| Mother-daughter communication  Conflict negotiation and  Effective communication | Passive, aggressive, and assertive communication w/role plays  Observed mother-daughter conflict discussion w/feedback; Mother-daughter values discussion; Rephrase it-game – Using I-statements | St,Si,Sa  St  St | W,G,J  J  J |
| Affective characteristics  Strengthen bond, increase  positive interactions  Mothers as resources for HIV  prevention | Get to Know You Game; Reverse role-plays (girls w/someone else’s mother)  Successful AA women game; Mother-daughter LIPSTICK  Public service announcement creation  Choosing assertive responses; Mother-daughter LIPSTICK  Mother-daughter values discussion; Mother challenge – scenario  Adolescent development | St  Si  St  Si  St  St | J  J  J  J  J  W |
| Peer and Partner Relationships |  |  |  |
| Partner sexual communication | KISS – Keep It Simple Sister; Comebacks to pressure lines  Passive, aggressive, and assertive communication  Role-play different types of communication | Si  St,Si,Sa  St,Si,Sa | W,G  W,G,J  W,G |
| Partner/relationship characteristics and power dynamics (including violence) | Pieces and parts of relationships, gender-role stereotypes  Healthy versus unhealthy relationships, concurrent partnerships  Choosing healthy relationships; What does abuse look like?  Partner selection/types (casual, serious) & implications for HIV risk | Si  Si  Si  Si | W,G  W,G  W,G  W |
| Peer Influences | Challenging peer norms, using the group to reinforce safer sex messages | St,Si,Sa | W,G,J |

# ^1^ Sa=Sista, Si=SiHLE, St=STYLE; ^2^ W=Women, G=Girls, J=Joint Mother-Daughter Dyad

IMARA is structured like Project STYLE. Separate mother and daughter groups cover parallel content and run simultaneously, and joint activities enhance mothers’ credibility as a resource for HIV/STI prevention, practice new communication skills, negotiate conflict, and strengthen the mother-daughter relationship. Activities reinforce the reciprocal impact of mothers and daughters, and enhance safe sex knowledge, attitudes, and skills. Morning and afternoon sessions begin with an icebreaker and/or poem to enhance ethnic and gender pride. IMARA’s goals and motto are presented to emphasize strong mother-daughter relationships, foster sisterhood, build group cohesion, and increase motivation. Ground rules are reviewed, and each woman and girl signs the IMARA pact to confirm her commitment to the program. At the end of day 1, mothers and daughters receive homework for the week. Woven throughout IMARA is the impact of alcohol and drug use on risk behavior, including condom use while high. Table 1 lists the theoretical constructs, intervention activities, target participant, and the original program from which the activity is drawn. Appendix 1 includes IMARA’s curriculum.

FUEL™: Health Promotion Control Group. Mothers and daughters randomly assigned to FUEL™ (N=150) will participate in separate 2-day workshops identical in length and intensity to IMARA. FUEL Health Promotion Control combines two programs, FUEL and Project Balance Health Promotion. FUEL™ promotes healthy activities by encouraging good nutrition, exercise, and informed consumer behavior. FUEL™ does not explicitly address HIV/STI prevention. However, the high prevalence of HIV/STIs among AA women and girls make it ethically questionable to withhold all information about HIV/STIs. Thus, we will present information from Balance’s session about HIV/AIDS, condoms, and other STIs. We will also insert the following sessions from Balance into FUEL: alcohol use, drug/marijuana use, nutrition, exercise, and violence to increase program length. FUEL™ is provided in Appendix 1.

Retention and Tracking Procedures

We will hire a retention coordinator to track subjects and address scheduling issues and barriers. She will telephone, text message, and/or e-mail families as appointment reminders. We will collect phone numbers, e-mail, and home addresses of at least three people who could help us locate them in the future. Based on prior experience, we will ask for contacts living in stable housing and family pastors. We will telephone, e-mail, and/or text families monthly to update our lists, and we will verify participants’ locating information each time we contact them. We will establish a toll-free number and project e-mail address. We will mail handwritten thank you cards after each interview/workshop to demonstrate our appreciation. We will send personalized birthday cards and give small gifts to sustain interest in the study. Two months after baseline, we will mail families a newsletter with fun facts about AA history and a list of activities or events around Chicago. We will include postage-paid address change cards with our toll-free number and e-mail address. If our correspondence is returned, we will telephone, e-mail, or text message families to obtain their new address. If we do not reach the family after one week and at least five attempts, we will contact people from participants’ records and use well-known internet locator services. We will send trackers to the family’s last known address and leave letters at homes asking families to call us. We will return to residences to ensure letters were received. Prior experience has taught us to ring all doorbells, visit homes after school, and wait for someone to return. If the family no longer lives at the address, we will ask the family’s contacts to help us locate them. At baseline, we will request permission to obtain forwarding information from girls’ schools.

Contact information will be requested from schools for tracking purposes only. We will use this approach to locate participants after we have exhausted all other contact information, including additional contacts, provided by the participants. Participants will be asked to sign a release of information form that we will use to request teen’s contact information from schools. We will send the school a letter on UIC letterhead, along with a copy of the release of information form indicating participant permission to release information to the research staff. The letter will not contain information about the study, only that the student is participating in a research study at UIC. We will call the school to follow-up if we do not hear back after sending the letter.

Procedures to Obtain Informed Consent/Assent

For both youth assent and parental permission and consent, we will use procedures used in multiple IRB-monitored studies at UIC. After an overview of the study is presented, an interviewer will review the informed consent and assent documents with mothers and daughters separately. Participants will be given the choice between reading the consent/assent documents themselves, or having the interviewer read it aloud to them. After reading the consent/assent document, follow-up questions will be asked in order to ascertain that participants understand the study and what they are agreeing to participate in. Girls’ refusal to participate will supersede mother’s consent.

The study recruiter, data manager, project coordinator, and/or research assistants will obtain parental consent and permission and youth assent. They will be trained on informed consent/assent procedures, sign a study confidentiality agreement, and complete CITI Human Subjects training, required by UIC.

Parental permission and assent will be obtained using a document in a language

understandable to participants and their parents.

At the beginning of the baseline assessment, the interviewer will review the informed consent and assent documents with mothers and daughters separately.

The informed consent and assent documents will be reviewed with mothers and daughters separatey to minimize the possibility of coercion and to facilitate open dialogue about the study (e.g., between study staff and participants).

Potential participants have several opportunities to decline receiving information about study and/or participation; when they are initially approached, during eligibility screening, and during informed consent/assent. Participants can withdraw from the study at any time without penalty.

As required by Federal regulations [CFR 46.408(a)] we will conduct formal assent with all teens, even if the parent allows the youth to participate. We will explain the nature of the project, and stress that participation is completely voluntary.

We will give participants the choice between reading the consent/assent documents themselves, or having the interviewer read it aloud to them. After reading the consent/assent document, follow-up questions will be asked in order to ascertain that participants understand the study and what they are agreeing to do.

In addition, after reading the assent form to participants, interviewers/recruiters will ask participants the following questions:

(1) What is the study about?

(2) How much time will it take to be in the study?

(3) What will I/my daughter be asked to do?

(4) Do you/does your daughter have to participate?

(5) Will your/your daughter’s treatment be different if you take part in the study?

(6) How can you/your daughter get out of the study if you decide not to be in it later?

(7) How is you/your daughter’s data kept confidential?

(8) What are the benefits of being in the study?

(9) What are the risks of being in the study?

(10) There are certain times that I can’t keep what you/your daughter say(s) private. What are some examples of those times?

Respondents who are able to, in the judgment of the interviewer, communicate and give acceptable answers to these questions will be considered eligible to assent/consent.

Whenever the interviewer/recruiter feels there is a question about the need for a more formal assessment of the decisional capacity of a potential participant he/she will be instructed to contact the PI or Project Coordinator before proceeding with the assent/consent procedures.

Potential participants will be informed that their decision to either participate or not participate will not impact their involvement with the community clinic recruitment sites and/or UIC or any future treatment they may seek or receive.

Only the signature of the participating mother will be solicited. We will not seek the consent of non-participating gaurdians/parents as they will not be involved in the study. We will ask the mother if she is the participating adolescent’s legal guardian.

The signed assent form will serve as documentation of assent. If the adolescent does not assent to the study, the interviewer/recruiter will notify the Project Coordinator. The Project Coordinator will keep track of the reasons why assent wasn’t granted if known.

The signed consent form will serve as documentation of permission. If the parent does not grant permission or consent to the study, the interviewer will notify the Project Coordinator. The Project Coordinator will keep track of the reasons why permission or consent wasn’t granted if known.

Parental permission will be obtained before research begins, before the first interview at UIC's IJR or COIP, or other convienent locations for families.

During the one year participation period some daughters will turn 18, we will re-consent these teens with a legally effective re-enrollment consent document using the same procedures outlined above.

If custody is transferred during the course of the research, and a new legal guardian is appointed to the participating adolescent, we will follow the following procedures to ensure the daughter’s continued participation in the study: a) We will obtain information regarding the new legal guardian from the former legal guardian or participating girl when we learn of the change in guardianship (e.g., telephone contact, scheduling the follow up assessment), b) Consistent with the above described procedures, we will verify the status of the new legal guardian through conversation with the participating girl and/or her former legal guardian, c) We will follow the same consent procedures as outlined above for the new legal guardian; however we will use the Legal Guardian Permission, Consent, and HIPAA Authorization For Adolescent Participation consent form. The former guardian initially completes the baseline assessment and two intervention workshops with the daughter. If guardianship changes following the baseline assessment but prior to the intervention workshop, we will identify and invite the new female guardian to participate in the study. In this case, the initial guardian will no longer be a participant because she did not attend the intervention workshops.

If, following the intervention workshops, guardianship changes, we will continue to collect the 6- and 12-month follow up assessments on the former guardian to examine her behavior change. The new guardian will not be an active participant in the study and no data will be collected from her because she did not participate in the intervention workshops. Rather, we will only request her permission to allow the adolescent to continue to participate in the later stages of the study (e.g., tracking, retention, follow up assessments). The former legal guardian will still remain actively enrolled in the research; she will continue her participation in the follow-up assessments and the data previously collected will be retained.

Risks

The risks associated with this study are minimal and mainly concern (a) a potential breach of confidentiality, and/or (b) discomfort with the assessment questions and intervention materials. Women and girls may feel uncomfortable answering the questions or the assessment procedures (e.g., using a computer), they may get upset when sensitive topics come up during the intervention sessions, and they may experience discomfort talking to each other about difficult topics. Participants may get tired of answering the questionnaires. They may feel concerned that other group members will tell confidential information to outsiders. Strategies to minimize these risks are described below. Lastly, if a subject discloses abuse or significant risk of harm to self or others, our staff is mandated to report these concerns and will have to inform the appropriate authorities. Participants are clearly informed of these exceptions to confidentiality. Of note, most of the measures have been used extensively in clinical research with no known adverse effects. Likewise, no adverse effects have resulted from the HIV prevention interventions we have conducted to date.

Protection against risks

Protection against a breach of confidentiality

A number of procedures will be taken to minimize risk of a breach of confidentiality. First, to protect the integrity of the participants’ data, the Data Manager will assign all families a random code number. This code number will be used on all information collected from participants, including questionnaires and computerized structured interviews. Since the study is longitudinal, we will maintain lists of participants with links between identifying information and code numbers. Only the Principal Investigator, Co-Investigator, Project Coordinator, Project Recruiter, and Data Manager will have access to these lists, which are kept in locked and/or password protected files. Other study personnel will have access on an as needed basis to individual participants’ names and code numbers in order to adequately perform their duties, i.e., interviewers must label the questionnaires with the correct code number of the participant whom they are interviewing. All personnel will complete extensive training before they are granted access to this identifying information. They will complete the Human Subjects Training sponsored by the University of Illinois at Chicago, which complies with federal guidelines delineated in 45 CFR Part 46. Personnel will also sign confidentiality statements that specify that if the participants’ confidentiality is breached unintentionally that personnel will follow the procedures for reporting this breach to the Principal Investigator. The confidentiality statement also states that unintentional or deliberate violations of participants’ confidentiality may result in demotion or termination depending upon the severity of the event. Personnel will also participate in training with the Principal Investigator and/or Project Coordinator regarding data safety, confidentiality of participants, limits of confidentiality, and proper administration of the study protocol. We will store all hard copies of the data in locked cabinets and only study staff will have access to them. After completion of an interview with a study participant, data with code numbers will be placed in a file cabinet for data entry. Data that are entered into computer files will be password protected and maintained on a subdirectory of a server, maintained by the Community Outreach Intervention Projects (COIP). Only the Principal Investigator, Co-Investigator, Project Coordinator, Data Manager, and data entry assistants will have access to this subdirectory. The Principal Investigator will review all requests, current and future, to use the data, and any data files that are provided to other individuals will be stripped of identifiers. Data files will only contain code numbers so that data across multiple assessment waves can be matched. Research assistants with access to the data will be monitored very closely (e.g., no research materials will be allowed to leave the laboratory) and will be informed about the importance of confidentiality when they join the project and at laboratory meetings throughout the project. We will prepare written reports without any identifiable information about specific girls and mothers. Participants will be asked to provide a code word when they submit their urine sample for testing. This code word will be unique to them and something they will remember. This code word will be described to the participants as being: a word that only they will know and that other family members, including their mother/daughter, will not know, a word that the participant will be sure to remember, and a word that they will not share with anybody else. If participants ask for guidance in choosing a code word, we will suggest that participants might use the name of their favorite actor, musician, or favorite book. We will stress that participants are not to share their code word with their mother/daughter and the word should not be a word that is easily identifiable by anyone else. This code word, in addition to participants’ full name and date of birth, will then be used to verify identity and as an extra precaution to maintain confidentiality when disclosing STI test results.

Second, we will apply for a Certificate of Confidentiality, and the certificate will be maintained through the life of the research project. We will inform study participants of the above procedures and the limits of confidentiality during the consent/assent process at the beginning of the interview. Specifically, we will warn participants that state law mandates reporting of abuse and/or neglect of children, and that threat of harm to self or others requires intervention by clinical staff. We will inform participants that criminal behavior i.e. drug use, is not reported to authorities, and that the security of this information is protected by the Certificate of Confidentiality.

Third, to guard against breaches of confidentiality during the tracking process, we will take the following steps to preserve confidentiality and ensure that information about the nature of the research project or the criteria for inclusion are not revealed to the girl’s school; a) In any contact with the school, research staff will be carefully trained not to disclose the nature of the research project or the eligibility criteria (i.e., some participants will be in psychiatric care), b) research staff will read a predetermined script, and c) written materials sent to the school will NOT include the name of the project or be printed on the COIP letterhead but will instead be printed on “University of Illinois at Chicago” letterhead without a specific department listed.

Fourth, the importance of confidentiality will be reviewed at the start of the intervention. At the beginning of the intervention, we will review the group expectations/rules which include the importance of confidentiality. Group members will be reminded throughout the two intervention days that what is said in the group should remain in the group. We have not experienced any adverse events related to breaches of confidentiality in any of our previous studies.

Protection against discomfort

With regard to the potential discomfort mothers and daughters may feel during the study, we will emphasize that their participation is completely voluntary. We will further explain that if any part of the study leads them to feel uncomfortable or uneasy they are free to discontinue it. Mothers and daughters will not be forced to participate in any aspect of the study, and they may choose not to answer questions or opt out of group activities. They need only tell the group leaders that they do not want to participate. In the unlikely event that a participant experiences considerable distress as a result of the procedures, or discloses abuse, neglect, or suicidal ideation or intent, research staff will advise the subject to discuss it with his/her therapist if they are still receiving mental health services, and she will be evaluated for potential imminent risk. Dr. Donenberg is a licensed clinical psychologist and will be on-call for any problems that may arise during the interviews or interventions. She will guide the interviewers and facilitators to assess participants’ level of risk and determine the appropriate course of action (e.g., send to the emergency room, referrals for treatment, shelters, hotline numbers).

The PI has extensive clinical procedures in place to address crisis situations, including in-depth training and consultation with an on-call psychiatric resident. These procedures have been used for over 12 years with no adverse events.

Benefits

There are several potential benefits to this study. 1) Mothers and daughters may enjoy the interventions and talking to a trained interviewer and facilitator about their concerns. 2) The interventions may reduce risk behavior and HIV transmission and/or acquisition. 3) Mothers and daughters may enjoy learning to communicate more effectively with each other, and this may have long term benefits for their relationship and their partner relationships. 4) Mothers and daughters may benefit from the opportunity to discuss and reflect on concerns related to sexual risk-taking. 5) The information gained from the study may be used to improve HIV prevention programs for youth and families in other countries and cultures.

Health disparities in HIV/AIDS/STIs and mental health among African American girls and women have continued to rise at an alarming rate over this decade. Mental health and HIV/AIDS are linked in important ways. The proposed multilevel intervention targets both high risk groups, women and their daughters, and it may reduce risky behaviors that expose them to HIV/AIDS. Hence, the benefits outweigh the potential risks. Even if a girl and/or mother does not directly benefit from the project, the information gained may be used to improve the effectiveness of HIV education and intervention for other families.

Maintaining the Confidentiality of Research Data

The study recruiter will be instructed to keep information provided by potential participants completely confidential unless the sensitive information is of a nature that is required by law to be reported to law enforcement authority (e.g., incidents of abuse or incest). All study staff will sign a confidentiality agreement.

No process or outcome data will contain personal identifying information. Each participant will be assigned a unique identification number (identifier) that will be associated with all evaluation data so that no names are ever associated with specific responses. The only exception is if a participant tests positive for an STI, we are required by law to report positive STI case by name and contact information to the participant’s local health department. The UIC adolescent medicine physician or her associate will have access to UIC clinic records of those subjects who choose to be treated by her. Participants will be asked to provide a code word when they submit their urine sample for testing. This code word will be unique to them and something they will remember. This code word will be described to the participants as being: a word that only they will know and that other family members, including their mother/daughter, will not know, a word that the participant will be sure to remember, and a word that they will not share with anybody else. If participants ask for guidance in choosing a code word, we will suggest that participants might use the name of their favorite actor, musician, or favorite book. We will stress that participants are not to share their code word with their mother/daughter and the word should not be a word that is easily identifiable by anyone else. This code word, in addition to participants’ full name and date of birth, will then be used to verify identity and as an extra precaution to maintain confidentiality when disclosing STI test results.

Tracking and demographic information will be collected and kept separate from assessment data. This information will be stored in a locked filing cabinet accessible only by the research team. We will maintain lists of participants with links between identifying information and code numbers. Only the Principal Investigator, Project Coordinator, Project Recruiter, and Data Manager will have access to these files, which are kept in locked or password protected files.

This is a longitudinal study, therefore names, dates, and contact information will be collected to assist in tracking each participant. This information will be kept separate from the interview/questionnaire data we collect.

Interview/questionnaire data will be coded with a code number, and stored in a password protected folder on a secure server and on a secure password protected computer. Identifying information will be stored separate from the interview/questionnaire data in a secure password protected folder on a secure server and on a secure password protected computer.

A master list linking the code number with participant name will be stored on a password protected computer and in a password protected folder on a secure server. The server will be maintained by COIP. Only the PI, CoI, Project Coordinator, Recruiter, and Data Manager will have access to these files.

Any paper-pencil interview/questionnaire data collected will be stored in a locked office in a locked filing cabinet, and coded with a code number, not an identifer. Urine samples for STI testing will be coded with a code number and stored temporarily in a locked office at UIC.

Data will be stored on a password protected computer, in a locked office, on a password protected folder on a secure network maintained by COIP, hard copy data will be stored in locked cabinets in a locked office, urine samples will be temporarily stored in a locked office. Data containing non-identifiable data will be temporarily stored on password protected laptops.

We will destroy all identifiers at the end of the study once all data is organized and datasets are cleaned.

This information will be included in the consent and assent documents and written consent/assent will be received for all particiapants.

To guard against breaches of confidentiality during the tracking process, we will take the following steps to preserve confidentiality and ensure that information about the nature of the research project or the criteria for inclusion are not revealed to the girl’s school; a) In any contact with the school, research staff will be carefully trained not to disclose the nature of the research project or the eligibility criteria, b) research staff will read a predetermined script, and c) written materials sent to the school will NOT include the name of the project or be printed on the COIP letterhead but will instead be printed on “University of Illinois at Chicago” letterhead without a specific department listed.

Data Safety Monitoring Plan

Anticipated adverse events include participant discomfort and the need to violate participant confidentiality. All study personnel will be trained regarding the limits of confidentiality. Training will include reviewing possible scenarios and knowledge of key questions to assess level of risk. We will train interview staff to err on the side of caution and to contact a clinical supervisor as needed. Supervisors will be available by phone 24-hours a day should interviewers need to consult regarding an emergency. In this situation, we will train interviewers to leave participants in the company of study personnel and immediately contact clinical supervisors before participants leave the interview room. Under the guidance of clinical supervisors, interviewers will be trained to contact University Police to ensure the safety of participants should they report imminent risk for suicide or abuse (i.e., taken to the emergency room).

A number of procedures will be taken to minimize risk of a breach of confidentiality. First, to protect the integrity of the participants’ data, the Data Manager will assign all families a random code number. This code number will be used on all information collected from participants, including questionnaires and computerized structured interviews. Since the study is longitudinal, we will maintain lists of participants with links between identifying information and code numbers. Only the Principal Investigator, Co-Investigator, Project Coordinator, Project Recruiter, and Data Manager will have access to these lists, which are kept in password protected and locked files. Other study personnel will have access on an as needed basis to individual participants’ names and code numbers in order to adequately perform their duties, i.e., interviewers must label the questionnaires with the correct code number of the participant whom they are interviewing. All personnel will complete extensive training before they are granted access to this identifying information. They will complete the Human Subjects Training sponsored by the University of Illinois at Chicago, which complies with federal guidelines delineated in 45 CFR Part 46. Personnel will also sign confidentiality statements that specify that if the participants’ confidentiality is breached unintentionally that personnel will follow the procedures for reporting this breach to the Principal Investigator. The confidentiality statement also states that unintentional or deliberate violations of participants’ confidentiality may result in demotion or termination depending upon the severity of the event. Personnel will also participate in training with the Principal Investigator and/or Project Director regarding data safety, confidentiality of participants, limits of confidentiality, and proper administration of the study protocol. We will store all hard copies of the data in locked cabinets and only study staff will have access to them. After completion of an interview with a study participant, data with code numbers will be placed in a file cabinet for data entry. Data that are entered into computer files will be password protected and maintained on a subdirectory of a server, maintained by COIP. Only the Principal Investigator, CoI, Project Coordinator, Data Manager, Recruiter, and data entry assistants will have access to this subdirectory (it is password protected). Participants will be asked to provide a code word when they submit their urine sample for testing. This code word will be unique to them and something they will remember. This code word will be described to the participants as being: a word that only they will know and that other family members, including their mother/daughter, will not know, a word that the participant will be sure to remember, and a word that they will not share with anybody else. If participants ask for guidance in choosing a code word, we will suggest that participants might use the name of their favorite actor, musician, or favorite book. We will stress that participants are not to share their code word with their mother/daughter and the word should not be a word that is easily identifiable by anyone else. This code word, in addition to participants’ full name and date of birth, will then be used to verify identity and as an extra precaution to maintain confidentiality when disclosing STI test results.

The Principal Investigator will review all requests, current and future, to use the data, and any data files that are provided to other individuals will be stripped of identifiers. Data files will only contain code numbers so that data across multiple assessment waves can be matched.

Research assistants with access to the data will be monitored very closely and will be informed about the importance of confidentiality when they join the project and at laboratory meetings throughout the project. We will prepare written reports without any identifiable information about specific girls and mothers.

Second, we will apply for a Certificate of Confidentiality, and the certificate will be maintained through the life of the research project. We will inform study participants of the above procedures and the limits of confidentiality during the consent/assent process at the beginning of the interview. Specifically, we will warn participants that state law mandates reporting of abuse and/or neglect of children, and that threat of harm to self or others requires intervention by clinical staff. We will inform participants that criminal behavior i.e. drug use, is not reported to authorities, and that the security of this information is protected by the Certificate of Confidentiality.

Third, to guard against breaches of confidentiality during the tracking process, we will take the following steps to preserve confidentiality and ensure that information about the nature of the research project or the criteria for inclusion are not revealed to the girl’s school; a) In any contact with the school, research staff will be carefully trained not to disclose the nature of the research project or the eligibility criteria, b) research staff will read a predetermined script, and c) written materials sent to the school will NOT include the name of the project or be printed on the COIP letterhead but will instead be printed on “University of Illinois at Chicago” letterhead without a specific department listed.

Fourth, the importance of confidentiality will be reviewed at the start of the intervention. At the beginning of the intervention, we will review the group expectations/rules which include the importance of confidentiality. Group members will be reminded throughout the two intervention days that what is said in the group should remain in the group. We have not experienced any adverse events related to breaches of confidentiality in any of our previous studies.

With regard to the potential discomfort mothers and daughters may feel during the study, we will emphasize that their participation is completely voluntary. We will further explain that if any part of the study leads them to feel uncomfortable or uneasy they are free to discontinue it. Mothers and daughters will not be forced to participate in any aspect of the study, and they may choose not to answer questions or opt out of group activities. They need only tell the group leaders that they do not want to participate. In the unlikely event that a participant experiences considerable distress as a result of the procedures, or discloses abuse, neglect, or suicidal ideation or intent, research staff will advise the subject to discuss it with his/her therapist if they are still receiving mental health services, and she will be evaluated for potential imminent risk. Dr. Donenberg is a licensed clinical psychologist and will be on-call for any problems that may arise during the interviews or interventions. She will guide the interviewers and facilitators to assess participants’ level of risk and determine the appropriate course of action (e.g., send to the emergency room, referrals for treatment, shelters, hotline numbers). We have extensive clinical procedures in place to address crisis situations, including in-depth training and consultation with an on-call psychiatric resident. These procedures have been used for over 12 years with no adverse events.

Unanticipated adverse events will be brought to the attention of the Principal Investigator as soon as possible, and reported immediately to the University IRB. The IRB will determine whether it is appropriate to stop the study protocol temporarily or will provide suggestions/modifications to the study procedures. Possible modifications include adding possible adverse events to the consent form and re-consenting all study participants. The PI will be responsible for monitoring participant safety on a monthly basis at regularly scheduled research meetings. She will keep a written log of all adverse events and ensure that the IRB is contacted immediately. She will also keep a log of the outcome of IRB decisions regarding adverse events and apprise the research team of any changes that need to occur as a result.

Data Safety and Monitoring Board

In accordance with the National Institutes’ of Health requirement that clinical trials have Data and Safety Monitoring Boards (DSMB), we will establish a five-member DSMB according to policies and requirements put forth by NIH. The proposed study is considered to present minimal risk to participants given that subjects will complete questionnaires and participate in a psychosocial intervention.

DSMB Member Responsibilities: Members of the DSMB will perform the following activities: a) Review the research protocol and plans for data and safety monitoring. b) Review progress of the trial, including analysis of data quality and timeliness; subject recruitment, randomization and retention; subject risk versus benefit; and other factors that may affect outcome. c) Review serious adverse event reports, provide commentary, and provide oversight to ensure that reports are relayed to individual IRBs and to the Office of Human Research Protections (OHRP), as indicated. d) Review analyses of outcome data of the proposed study and review reports of related studies to determine whether the proposed study needs to be changed or terminated. e) Determine whether the trial should continue as designed, should be changed, or should be terminated based on the data and make recommendations to NIH, Institutional Review Boards, and investigators considering conclusion or continuation of the study. f) Review proposed modifications to the study prior to their implementation. g) Protect the confidentiality of the trial data and the results of the monitoring. h) Determine whether and to whom outcome results should be released prior to the reporting of study results. i) Following DSMB meetings, provide appropriate NIH staff and the PI with written information concerning their findings.

DSMB Membership: The DSMB members will be chosen by Dr. Donenberg. All members will have voting rights and will be chosen based upon their knowledge of clinical trial methodology, their experience, and absence of conflicts of interest. They will be appointed for the life of the project. The Chair of the DSMB will be selected from among the DSMB members.

DSMB Meetings: DSMB meetings will be held every twelve months beginning in Year 2 of the study. However, the Chair of the DSMB can call a meeting as needed. Serious adverse events will be reported to the Chair as soon as they occur. The Chair of the DSMB will determine whether an in-person meeting or teleconference is needed. Prior to the meetings, a written report containing any outcome data will be sent to DSMB members by the study statistician. Each meeting will be divided into three parts. (1) There will be an open session in which the PI will review the progress of the study and answer questions from members of the DSMB. (2) A closed session involving the DSMB members and the study statistician will be held during which outcome results will be discussed. (3) A final session involving only the DSMB members will be held to discuss the progress of the study and the outcome results, to develop recommendations, and to takes votes as necessary.

DSMB Recommendations: The DSMB recommendations will be given to the PI. If the DSMB recommends a change to the study or that a study be closed, the PI will act as quickly as possible upon those recommendations. If the PI disagrees with the DSMB, the NIH Project Officer will be notified and all parties will reach a mutually acceptable decision. Confidentiality will be maintained throughout this process unless relevant data need to be presented in order to reach a mutually acceptable decision.

Release of Outcome Data: Outcome data will not be made available to individuals outside of the DSMB. Each member of the DSMB will sign a statement of confidentiality.

Conflict of Interest: Members of the DSMB will disclose any potential conflicts of interest, either pre-existing or those that develop during their tenure, to the PI and the NIH Project Officer.

Data Analyses

We will evaluate the effect of IMARA compared to a health promotion control group (FUELTM) in reducing risky sexual behaviors among 300 AA or black women and their daughters. Mother-daughter dyads will be randomly assigned to IMARA (n1=150) or FUELTM (n2=150). Measures of treatment success will be collected at three visits (baseline, 6-, and 12-months). Preliminary analyses will examine whether those who complete the study differ systematically on baseline data from non-completers using chi-square or t-tests. The primary analyses will include all subjects in an intent-to-treat design (see Missing Data below) to evaluate treatment effects and to compare the impact of hypothesized mediators (individual attributes, family context, relationship concerns) and moderators on treatment efficacy. Treatment outcomes will be analyzed using logistic and linear multiple regression models for categorical and continuous outcomes, respectively, examining effects at 6- and 12- months separately as well as a combined model with random effects for repeated measurements across time.

Data reduction and preliminary analyses. We will create summary scores for (1) mother-daughter relationship (warmth and control), (2) mother-daughter communication (quality and quantity), (3) maternal behavior and attitudes (sexual behavior, partner relationships, relationship history), and (4) girls’ peer and partner relationships (maintenance concerns, peer influence, communication quality and quantity, self-efficacy). We will create two forms of the dependent variable separately for women and girls, a composite score comprised of multiple risk indicators and a set of individual indicators (e.g., non-condom use, number of partners). We will compare groups on baseline variables using ANOVAs or nonparametric Wilcoxon Rank-Sum tests (continuous) and Chi-square tests (categorical) and control for predictor variables in subsequent regression analyses that are not balanced by baseline randomization.

General statistical issues. We will conduct multiple comparisons as some outcomes may be correlated. We will control for Type I error in exploratory but not primary analyses in order to capture important findings that may be obscured from the conservative limits imposed by Bonferroni corrections. We will explore whether girls’ and women’s sexual behaviors are related to potentially important covariates (e.g., trauma history, pubertal status) and use regression models to test their effects. A key covariate analysis will evaluate the reciprocal impact of mothers and daughters on each other over time. To evaluate the impact of mothers on daughters, we will include mothers’ characteristics as covariates in the regression model for daughter’s outcomes. We will use a similar approach to evaluate impact of daughters on mothers over time. Missing data will most likely occur as a result of subject attrition. Based on our intervention studies, we expect 85%-90% retention, in both arms. We will evaluate associations between dose (1 vs. 2 sessions) and efficacy. Missingness complicates statistical analyses via biased parameter estimates, reduced statistical power, and degraded confidence intervals. We expect minimal missingness in the data collection based on our prior research, but we will consider item and unit nonresponse. An analysis using only completers is generally valid assuming data are missing completely at random,332 but is inefficient because it discards data points observed for noncompleters. We will use all available data and produce valid inferences under the broader assumption that data are missing at random. We will quantify the potential bias of these inferences should we suspect missing data to be nonrandom.333, 334

Sample size and power considerations. Power analyses determined the sample size needed to compare IMARA vs. FUELTM in reducing risky sexual behavior. Outcomes are both continuous and categorical, but the latter present the greatest challenge to statistical power. Thus, we evaluated power using a dichotomous outcome (condom use at last sex – yes/no) at three levels of retention (70%, 80%, 90%) over 12- months. We calculated power to detect the effect of IMARA vs. FUELTM as a predictor of condom use at last intercourse, using a 2-sided test with alpha=.05, and assuming 20% of mothers and 50% of daughters in the control group will have used condoms at last intercourse. For daughters, we assume 50% will be sexually active at baseline, and an additional 20% will initiate sex during follow-up, such that 70% of the original sample will be available for analysis. Table 3 indicates power at different retention rates. Effect size (h) is a function of two proportions defined by Cohen;335 h=.2 is a small effect, h=.5 is a medium effect, and h=.8 is a large effect. For mothers, we will achieve 80% power to detect small to medium effects of h=.39, h=.36, and h=.34, and for daughters h=.46, h=.43, and h=.41, under retention rates of 70%, 80%, and 90%, respectively. We also

**Table 3. Power Analysis for Condom Use at Last Intercourse, 12 Months After Intervention** effect size (d) for

calculated the standard group mean comparisons with 80% power for continuous outcomes, where d=0 is defined as no treatment effect. Assuming 2-tailed tests and alpha=.05, we will be able to detect a small to medium effect size (d=0.39) even with 70% retention. For comparison of the proportion of daughters who initiate sex by the end of the study, we assume that of the 50% non-sexually active at baseline (n=75), 20% of the original sample (n=30, or p=30/75=.40) will initiate sex in the control group. We will have 80% power to detect a medium effect size under 70% retention (h=.48).

| Retention at 12 Months | Effect Size | Women: 150 in each group | | | | Effect Size | Daughters: 105 in each group sexually active | | | |
| --- | --- | --- | --- | --- | --- | --- | --- | --- | --- | --- |
|  |  | N per group at follow-up | Condom use in FUEL | Condom use in IMARA | Power |  | N per group at follow-up | Condom use in FUEL | Condom use in IMARA | Power |
| 70% | h=.28 | 105 | 20% | 32% | 50.9% | h=.41 | 73 | 50% | 70% | 70.6% |
|  | h=.36 | 105 | 20% | 36% | 73.6% | h=.64 | 73 | 50% | 80% | 97.3% |
|  | h=.44 | 105 | 20% | 40% | 89.1% | h=.93 | 73 | 50% | 90% | >99.9% |
| 80% | h=.26 | 120 | 20% | 32% | 56.4% | h=.41 | 84 | 50% | 70% | 75.9% |
|  | h=.36 | 120 | 20% | 36% | 79.2% | h=.64 | 84 | 50% | 80% | 98.7% |
|  | h=.44 | 120 | 20% | 40% | 92.7% | h=.93 | 84 | 50% | 90% | >99.9% |
| 90% | h=.26 | 135 | 20% | 32% | 61.4% | h=.41 | 94 | 50% | 70% | 80.0% |
|  | h=.36 | 135 | 20% | 36% | 83.7% | h=.64 | 94 | 50% | 80% | 99.3% |
|  | h=.44 | 135 | 20% | 40% | 95.2% | h=.93 | 94 | 50% | 90% | >99.9% |

**Table 4. Power analysis: Effect sizes at 80% power**

| Retention at 12 months | N per group at 12 months | Mean difference for continuous outcomes (SDs) |
| --- | --- | --- |
| 70% | n1=n2=105 | d=.39 |
| 80% | n1=n2=120 | d=.36 |
| 90% | n1=n2=135 | d=.34 |

Testing the specific aims to conduct a 2-arm RCT to compare IMARA vs. FUELTM on risky sexual behavior and to examine theoretical mediators. We will evaluate treatment effects separately at 6- months and at 12-months. We will also combine models to evaluate an average effect across both time points and to analyze change from 6- to 12-months. We will analyze binary outcomes using logistic regression and continuous outcomes using linear regression. At a single time point, we will test the effect of the binary indicator for IMARA vs. FUELTM on the treatment outcome, adjusting for confounders and the outcome at baseline, as additional independent variables. Models combining time points will include individual random effect terms to account for correlation within repeated measurements. We will test an average effect across time points and effects on patterns of change from 6- to 12- months using interactions with time. We will use survival analysis to examine sexual initiation over the course of the study. We hypothesize that non-sexually experienced girls in IMARA will, on average, initiate sex later than FUELTM participants. We will calculate the Kaplan-Meier survival curves419 and conduct long-rank tests to compare the two survival curves. We will use Cox regression and its extensions (e.g. Cox's Proportional Hazard Model with Time-Dependent Covariates) to test for other factors such as age or SES.

Mediators of treatment outcomes. We will evaluate the effects of IMARA on hypothesized mediators of sexual risk over 12-months using Baron and Kenny’s approach: (1) We will test whether the independent variables are related to the dependent variables. (2) We will assess whether the independent variables are linked to the mediator(s). (3) We will examine whether the mediator is associated with the dependent variables when the independent variable is controlled. We will use Wald-type tests of fitted coefficients calculated using the modified sandwich variance estimator (adjusted for multiple observations per person) to determine whether these criteria are satisfied. An absence of treatment effects may be clarified by the mediation analyses.

Moderation of treatment outcomes. Treatment effects may differ systematically among girls and mothers depending on a variety of factors, such as trauma history or pubertal status. Consistent with Baron & Kenny336 and Cohen & Cohen,335 we will evaluate the interactions between treatment condition and the moderators using the GLMM.

Literature Cited

1. Centers for Disease Control and Prevention. HIV/AIDS Surveillance Report, 2004: U.S. Department of Health and Human Services, Centers for Disease Control and Prevention; 2005.

2. National Center for Health Statistics. Health, United States, 2007 with Chartbook on Trends in the Health of Americans. Hyattsville, MD; 2007.

3. The Henry J. Kaiser Family Foundation. HIV/AIDS Policy Fact Sheet: African Americans and HIV/AIDS. 2008 vol. Washington DC; 2006.

4. Centers for Disease Control and Prevention. HIV/AIDS Surveillance Report, 2006, Vol. 18. In: U.S. Department of Health and Human Services, ed. Atlanta: Centers for Disease Control and Prevention; 2006.

5. Centers for Disease Control and Prevention. HIV/AIDS Surveillance in Women; 2006.

6. Miranda J, Green BL. The need for mental health services research focusing on poor young women. *The Journal of Mental Health Policy and Economics*. 1999;2:73-80.

7. United States Department of Health and Human Services. Mental Health: Culture, Race, and Ethnicity-Supplement to Mental Health: A Report of the Surgeon General. Rockville, MD: U.S. Department of Health and Human Services, Public Health Service, Office of the Surgeon General; 2001.

8. Carey MP, Carey KB, Kalichman SC. Risk for human immunodeficiency virus (HIV) infection among persons with severe mental illnesses. *Clinical Psychology Review*. 1997;17:271-291.

9. Carey MP, Carey KB, Weinhardt LS, Gordon CM. Behavioral risk for HIV infection among adults with a severe and persistent mental illness: Patterns and psychological antecedents. *Community Mental Health Journal*. 1997;33:133-142.

10. Donenberg G, Pao M. Youths and HIV/AIDS: Psychiatry's Role in a Changing Epidemic. *Journal of the American Academy of Child and Adolescent Psychiatry.* 2005;44:728-747.

11. Brown LK, Danovsky MB, Lourie KJ, DiClemente RJ, Ponton LE. Adolescents with psychiatric disorders and the risk of HIV. *Journal of the American Academy of Child and Adolescent Psychiatry*. 1997;36:1609-1617.

12. Donenberg GR, Emerson E, Bryant FB, Wilson H, Weber-Shifrin E. Understanding AIDS-risk behavior among adolescents in psychiatric care: Links to psychopathology and peer relationships. *Journal of the American Academy of Child Adolescent Psychiatry*. 2001;40:642-653.

13. Donenberg GR, Pao M. Youths and HIV/AIDS: Psychiatry's role in a changing epidemic. *Journal of the American Academy of Child and Adolescent Psychiatry*. 2005;44:728-747.

14. Institute of Medicine. *Unequal treatment: Confronting racial and ethnic disparities in health care*. Washington DC: The National Academies Press; 2003.

15. Office of Minority Health. Eliminate Disparities in Mental Health. 2006 vol: Centers for Disease Control and Prevention, Office of Minority Health; 1999.

16. Office of Minority Health. Eliminate disparities in HIV and AIDS. 2006 vol; 2006.

17. Centers for Disease Control and Prevention. Youth Risk Behavior Surveillance, 2003; 2004.

18. Taylor SE, Repetti RL. Health psychology: What is an unhealthy environment and how does it get under the skin? *Annual Review of Psychology*. 1997;48:411-447.

19. DiClemente RJ, Lodico M, Grinstead OA et al. African-American adolescents residing in high-risk urban environments do use condoms: Correlates and predictors of condom use among adolescents in public housing developments. *Pediatrics*. 1996;98:269-278.

20. Newman PAaZ, Marc A. Gender differences in HIV-related risk behavior among urban African American youth: A multivariate approach. *AIDS Education and Prevention,*. 2000;12:308-325.

21. Donenberg GR, Wilson H, Emerson E, Bryant FB. Holding the line with a watchful eye: The impact of perceived parental permissiveness and parental monitoring on risky sexual behavior among adolescents in psychiatric care. *AIDS Education and Prevention*. 2002;14:138-157.

22. Newman L. Epidemiology of STDs in African American Communities. *Consultation to address STD disparitites in African American Communities: Meeting Report*

Atlanta, Georgia: Division of STD Prevention, National Center of HIV/AIDS, Hepatitis, STD and TB Prevention, Centers for Disease Control and Prevention,

Department of Health and Human Services; 2007:7-8.

23. Centers for Disease Control and Prevention. STD Disparities Summary; 2010.

24. Stiffman AR, Cunningham R. The epidemiology of child and adolescent mental health disorders. In: Gibbs JT, ed. *Child and Adolescent Mental Health: Challlenges for Social Work Education and Practice*. Berkley, CA: University of California Press; 1991.

25. Treisman GJ, Angelino AF. *The psychiatry of AIDS: A guide to diagnosis and treatment*. Baltimore, MD: Johns Hopkins University Press; 2004:217.

26. Tubman JG, Gil AG, Wagner EF, Artigues H. Patterns of sexual risk behaviors and psychiatric disorders in a community sample of young adults. *Journal of Behavioral Medicine*. 2003;26:473-500.

27. Cournos F, McKinnon K, Wainberg M. What can mental health interventions contribute to the global struggle against HIV/AIDS? *World Psychiatry*. 2005;4:135-141.

28. Mehta S, Moore RD, Graham NMH. Potential factors affecting adherence with HIV therapy AIDS. *AIDS*. 1997;11:1665-1670.

29. Wagner GJ, Kanouse DE, Koegel P, Sullivan G. Adherence to HIV Antiretrovirals among persons with serious mental illness. In: Laurence J, ed. *Medication Adherence in HIV/AIDS*. Larchmont, NY: Mary Ann Liebert, Inc.; 2004:295-302.

30. Di Scipio WJ. Sex, drugs, and AIDS: Issues for hospitalized emotionally disturbed youth. *Psychiatric Quarterly*. 1994;65:149-155.

31. DiClemente RJ, Wingood GM. A randomized controlled trial of an HIV sexual risk-reduction intervention for young African-American women. *Journal of the American Medical Association*. 1995;274:1271-1276.

32. Wingood GM, DiClemente RJ. Application of the theory of gender and power to examine HIV-related exposures, risk factors, and effective interventions for women. *Health Education & Behavior*. 2000;27:539-565.

33. DiClemente RJ, Salazar L, Crosby R. A review of STD/HIV preventive interventions for adolescents: Sustaining effects using an ecological approach. *Journal of Pediatric Psychology*. 2007;32:888-906.

34. Sales JM, Milhausen RR, DiClemente RJ. A decade in review: Building on the experiences of past adolescent STI/HIV interventions to optimize future prevention efforts. *Sexually Transmitted Infection*. 2006:431-436.

35. Wyatt GE, Williams JK, Myers HF. African-American sexuality and HIV/AIDS: Recommendations for future research. *Journal of the National Medical Association*. 2008;100:44-51.

36. Centers for Disease Control and Prevention. HIV/AIDS among youth; 2006.

37. Centers for Disease Control and Prevention. Cumulative AIDS Cases; Exposure Categories;Ten States/Territories and Cities Reporting the Highest Number of AIDS Cases; International Statistics. 2001 vol: Centers for Disease Control; 2000.

38. Rosenberg PS, Biggar RJ. Trends in HIV incidence among young adults in the United States. *Journal of the American Medical Association*. 1998;279:1894-1899.

39. The Henry J. Kaiser Family Foundation. Illinois: Teen birth rate per 1,000 population by race/ethnicity, 2002. 2006 vol; 2002.

40. DiClemente RJ. Adolescents at risk for AIDS: AIDS epidemiology, and prevalence and incidence of HIV. In: Oskamp S, Thompson S, eds. *Understanding and Preventing HIV Risk Behavior*. Thousand Oaks, CA: Sage Publishing; 1996:13-30.

41. Pequegnat W, Szapocznik J. The role of families in preventing and adapting to HIV/AIDS: Issues and answers. In: Pequagnat W, Szapocznik J, eds. *Working with families in the Era of HIV/AIDS*. Thousand Oaks, CA: Sage Publications; 2000.

42. Centers for Disease Control and Prevention. HIV/AIDS and African American women: A consultation supporting CDC's heightened national response to the HIV/AIDS crisis among African Americans. Meeting Report. In: Centers for Disease Control and Prevention, National Center for HIV/AIDS VH, STD, and TB Prevention, Division of HIV/AIDS Prevention, eds. Atlanta, Georgia; 2007.

43. Centers for Disease Control and Prevention. HIV/AIDS among US women: Minority and young women at continuing risk. 2004 vol; 2000.

44. Illinois Department of Public Health. HIV/AIDS/STD monthly surveillance update: Demographic characteristics of cumulative STD cases 1/1/08 - 11/30/08 2008.

45. Hoberman HM. Ethnic minority status and adolescent mental health services utilization. *Journal of Mental Health Administration*. 1992;19:246-267.

46. Miranda J, Nakamura R, Bernal G. Including ethnic minorities in mental health intervention research: A practical approach to a long-standing problem. *Culture, Medicine and Psychiatry*. 2003;27:467-486.

47. Young AS, Klap R, Sherbourne CD, Wells KB. The quality of care for depressive and anxiety disorders in the United States. *Archives of General Psychiatry*. 2001;58:55-61.

48. Rodenborg NA. Services to African American children in poverty:: Institutional discrimination in child welfare? *Journal of Poverty*. 2004;8:109-130.

49. McKay M, Bell C. Constructing a children's mental health infrastructure using community psychiatry principles. *The Journal of Legal Medicine*. 2004;25:5-22.

50. Prather C, Fuller T, King W et al. Diffusing an HIV prevention intervention for African American women: Integrating Afrocentric components into the SISTA diffusion strategy. *AIDS Education and Prevention*. 2006;18:149-160.

51. Harvey SM. New kinds of data, new options for HIV prevention among women: A public health challenge. *Health Education & Behavior*. 2000;27:566-569.

52. Sanders-Phillips K. Factors influencing HIV/AIDS in women of color. *Public Health Reports*. 2002;117:S151-S156.

53. Wingood GM, DiClemente RJ. Partner influences and gender-related factors associated with noncondom use among young adult African American women. *American Journal of Community Psychology*. 1998;26:29-51.

54. Adimora AA, Schoenbach VJ, Martinson FE, Coyne-Beasley T, Dohorty I, Stancil TR. Heterosexually transmitted HIV infection among African Americans in North Carolina. *Journal of Acquired Immune Deficiency Syndromes*. 2006;41:616 - 623.

55. Perrino T, Fernandez M, Bowen S, Arheart K. Low-Income African American women's attempts to convince their main partner to use condoms. *Cultural Diversity & Ethnic Minority Psychology*. 2006;12:70 - 84.

56. Cicchetti D, Toth S, Maughan A. An Ecological-Transactional Model of child maltreatment. *Handbook of Developmental Psychopathology*; 2000:689 - 722.

57. Boyer CB, Kegeles SM. AIDS risk and prevention among adolescents. *Social Science and Medicine*. 1991;33:11-23.

58. Amaro H. Love, sex, and power: Considering women's realities in HIV prevention. *American Psychologist*. 1995;50:437-447.

59. O'Leary A, Wingood G. Interventions for sexually active heterosexual women. In: Peterson J, DiClemente R, eds. *Handbook of HIV Prevention*. New York: Kluwer Academic/Plenum Publishers; 2000:179-200.

60. Kirby D, Short L, Collins J et al. School-based programs to reduce sexual risk behaviors: A review of effectiveness. *Public Health Reports*. 1994;109:339-360.

61. Fantasia H. Concept analysis: Sexual decision-making in adolescence. *Nursing Forum*. 2008;43:80 - 90.

62. Frances RJ, Wilkstrom, T., et al. Contracting AIDS as a means of committing suicide. *American Journal of Psychiatry,*. 1985;142:656.

63. Kipke MD, Boyer C, Hein K. An evaluation of an AIDS risk reduction education and skills training (ARREST) program. *Journal of Adolescent Health*. 1993;14:533-539.

64. Henggeler SW, Melton GB, Rodrigue JR. *Pediatric and adolescent AIDS: Research findings from the social sciences*. Newbury Park, CA: Sage Publications; 1992.

65. DiClemente RJ, Brown LK, Beausoleil NI, Lodico M. Comparison of AIDS knowledge and HIV-related sexual risk behaviors among adolescents in low and high AIDS prevalence communities. *Journal of Adolescent Health*. 1993;14:231-236.

66. DiClemente RJ, Ponton LE, Hansen WB. New directions for adolescent risk prevention and health promotion research and interventions. In: DiClemente RJ, Hansen WB, Ponton LE, eds. *Handbook of Adolescent Health Risk Behavior*. New York City: Plenum Press; 1996:413-420.

67. Hingson R, Strunin L, Berlin B. Acquired immunodeficiency syndrome transmission: Changes in knowledge and behaviors among teenagers, Massachusetts statewide surveys, 1986 to 1988. *Pediatrics*. 1990;85:24-29.

68. Sikand A, Fisher M, Friedman SB. AIDS knowledge, concerns, and behavioral changes among inner-city high school students. *Journal of Adolescent Health*. 1996;18:325-328.

69. Hingson RW, Strunin L, Berlin BM, Heeren T. Beliefs about AIDS, use of alcohol and drugs, and unprotected sex among Massachusetts adolescents. *American Journal of Public Health*. 1990;80:295-299.

70. Keller SE, Schleifer SJ, Bartlett JA, Johnson RL. The sexual behavior of adolescents and risk of AIDS. *JAMA*. 1988;260:3586.

71. Rolf J, Nanda J, Baldwin J, Chandra A, Thompson L. Substance misuse and HIV/AIDS risks among delinquents: A prevention challenge. *The International Journal of the Addictions*. 1991;25:533-559.

72. Aruffo J, Gottlieb A, Webb R, Neville B. Adolescent psychiatric inpatients: Alcohol use and HIV risk-taking behavior. *Psychosocial Rehabilitation Journal*. 1994;17:150-156.

73. Mustanski B, Donenberg G, Emerson E. I can use a condom, I just don't:The importance of motivation to prevent HIV in adolescents seeking psychiatric care. *AIDS Behavior*. 2006;10:753-762.

74. Donenberg GR, Moss Schwartz R, Emerson E, Wilson HW, Bryant FB, Coleman G. Applying a cognitive-behavioral model of HIV risk to youths in psychiatric care. *AIDS Education and Prevention*. 2005;17:200-216.

75. Fisher WA, Fisher JD. Understanding and promoting AIDS-preventative behavior: Insights from the theory of reasoned action. *Health Psychology*. 1995;14:255-264.

76. Santelli JS, Kaiser J, Hirsch L, Radosh A, Simkin L, Middlestadt S. Initiation of sexual intercourse among middle school adolescents: The influence of psychosocial factors. *Journal of Adolescent Health*. 2004;34:200-208.

77. Sterk C, Klein H, Elifson K. Predictors of condom-related attitudes among at-risk women. *Journal of Women's Health*. 2004;13:676-688.

78. Lawrence J, Eldridge G, Reitman D, Little C, Shelby M, Brasfield T. Factors influencing condom use among African American women: Implications for risk reduction interventions. *American Journal of Community Psychology*. 1998;26:7-28.

79. Katz RC, Mills K, Singh NN, Best AM. Knowledge and attitudes about AIDS: A comparison of public high school students, incarcerated delinquents, and emotionally disturbed adolescents. *Journal of Youth and Adolescence*. 1995;24:117-131.

80. McFarlane AH, Bellissimo A, Norman GR. The role of family and peers in self-efficacy: Links to depression in adolescence. *American Journal of Orthopsychiatry*. 1995;65:402-410.

81. Koen L, Uys S, Niehaus D, Emsley R. Negative symptoms and HIV/AIDS risk-behavior knowledge in schizophrenia. *Psychosomatics*. 2007;48:128-134.

82. Meade CS, Sikkema KJ. HIV risk behavior among adults with severe mental illness: A systematic review. *Clinical Psychology Review*. 2005;25:433-457.

83. McKinnon K, Cournos F, Herman R. HIV among people with chronic mental illness. *Psychiatric Quarterly*. 2002;73:17-31.

84. DiClemente RJ, Ponton LE. HIV-related risk behaviors among psychiatrically hospitalized adolescents and school-based adolescents. *American Journal of Psychiatry*. 1993;150:324-325.

85. Johnson S, Cunningham-Williams R, Cottler L. A tripartite of HIV-risk for African American women: The intersection of drug use, violence, and depression. *Drug and Alcohol Dependence*. 2003;70:169-175.

86. Shrier LA, Harris SK, Sternberg M, Beardslee WR. Associations of depression, self-esteem, and substance use with sexual risk among adolescents. *Preventive Medicine*. 2001;33:179-189.

87. Brooks-Gunn J, Paikoff R. Sexuality and developmental transitions during adolescence. In: Schulenberg J, Maggs JL, Hurrelmann K, eds. *Health Risks and Developmental Transitions During Adolescence*. New York: Cambridge University Press; 1997:190-219.

88. Rotheram-Borus MJ, Koopman, C., et al. Barriers to successful AIDS prevention programs with runaway youth. In: Woodruff J, Doherty, D., et al, ed. *Troubled Adolescents and HIV Infection: Issues in Prevention and Treatment*. Washington, DC: Georgetown University; 1989.

89. Whitbeck LB, Conger RD, Kao M-Y. The influence of parental support, depressed affect, and peers on the sexual behaviors of adolescent girls. *Journal of Family Issues*. 1993;14:261-278.

90. Dolcini MM, Adler NE. Perceived competencies, peer group affiliation, and risk behavior among early adolescents. *Health Psychology*. 1994;13:496-506.

91. Hayes CD, ed. *Risking the future: Adolescent sexuality, pregnancy, and childbearing*. Washington D.C.: National Academy Press; 1987;1.

92. Rotheram-Borus MJ, Rosario M, Reid H, Koopman C. Predicting patterns of sexual acts among homosexual and bisexual youth. *American Journal of Psychiatry*. 1995;152:588-595.

93. Perkins DF, Luster T, Villarruel FA, Small S. An ecological, risk-factor examination of adolescents' sexual activity in three ethnic groups. *Journal of Marriage and the Family*. 1998;60:660-673.

94. Bachanas PJ, Morris MK, Lewis-Gess JK et al. Psychological adjustment, substance use, HIV knowledge, and risky sexual behavior in at-risk minority females: Developmental differences during adolescence. *Journal of Pediatric Psychology*. 2002;27:373-384.

95. Cole PM, Michel MK, Teti LO. The development of emotion regulation and dysregulation: A clinical perspective. *Monographs of the Society for Research in Child Development*. 1994;59:73-100.

96. Sheeber L, Hops, H., et al. Family processes in adolescent depression. *Clinical Child and Family Psychology Review*. 2001;4:19-35.

97. Sheeber L, Allen, N., et al. Regulation of negative affect during mother-child problem-solving interactions: Adolescent depressive status and family processes. *Journal of Abnormal Child Psychology*. 2000;28:467-479.

98. Fritsch S, Donaldson, D., et al. Personality characteristics of adolescent suicide attempters. *Child Psychiatry and Human Development,*. 2000;29:219-235.

99. MacLean MG, Paradise, M.J., et al. Substance use and psychological adjustment in homeless adolescents: A test of three models. *American Journal of Community Psychology,*. 2000;27:405-427.

100. DiClemente RJ, Wingood GM, Crosby RA et al. A perspective study of psychological distress and sexual risk behavior among Black adolescent females. *Pediatrics*. 2001;108:1-6.

101. Phinney JS. Understanding ethnic diversity: The role of ethnic identity. *American Behavioral Scientist*. 1996;40:143-152.

102. Townsend TG. The impact of self-components on attitudes towards sex among African American preadolescent girls: The moderating role of menarche. *Sex Roles*. 2002;47:11-20.

103. Salazar LF, DiClemente RJ, Wingood GM et al. Self-concept and adolescents' refusal of unprotected sex: A test of mediating mechanisms among African American girls. *Prevention Science*. 2004;5:137-149.

104. Locke T, Newcomb M. Correlates and predictors of HIV risk among inner-city African American female teenagers. *Health Psychology*. 2008;27:337 - 348.

105. McKay M, Bannon W, Rodriguez J, Chasse K. Understanding African American Youth HIV Knowledge: Exploring the Role of Racial Socialization and Family Communication About "Hard to Talk About Topics". In: McKay M, Paikoff R, eds. *Community Collaborative Partnerships*. New York: The Haworth Press, Inc.; 2007:81 - 97.

106. Beadnell B, Stielstra S, Baker S et al. Ethnic identity and sexual risk-taking among African-American women enrolled in an HIV/STD prevention intervention. *Psychology, Health & Medicine*. 2003;8:187-198.

107. Wingood GM, Hunter-Gamble D, DiClemente RJ. A pilot study of sexual communication and negotiation among young African American women: Implications for HIV prevention. *Journal of Black Psychology*. 1993;19:190-203.

108. Scott KD, Gilliam A, Braxton K. Culturally competent HIV prevention strategies for women of color in the United States. *Health Care for Women International*. 2005;26:17-45.

109. Andrinopoulos K, Kerrigan D, Ellen J. Understanding sex partner selection from the perspective of inner city black adolescents. *Perspect Sex Preprod Health*. 2006;38:132 - 138.

110. Purdie V, Downey G. Rejection sensitivity and adolescent girls' vulnerability to relationship-centered difficulties. *Child Maltreatment*. 2000;5:338-349.

111. Kerrigan D, Andrinopoulos D, Johnson R, Parham P, Thomas T, Ellen J. Staying strong: Gender ideologies among African-American adolescents and the implications for HIV/STI prevention *Journal of Sex Research*. 2007;44:172 - 180.

112. Jipguep M-C, Sanders-Phillips K, Cotton L. Another look at HIV in African American Women: The impact of psychosocial and contextual factors. *Journal of Black Psychology*. 2004;30:366-385.

113. Peterson JL, Catania JA, Dolcini MM, Faigeles B. Multiple sexual partners among blacks in high-risk cities. *Family Planning Perspectives*. 1993;25:263-267.

114. Zelnik M, Shah SK. First intercourse among young americans. *Family Planning Perspectives*. 1983;15:64-70.

115. Begley E, Crosby RA, DiClemente R, Wingood G, Rose E. Older partners and STD prevalence among pregnant African American teens. *Sexually Transmitted Diseases*. 2002;30.

116. Center for AIDS Prevention Studies. What are young women's HIV prevention needs?: Center for AIDS Prevention Studies (CAPS) and the AIDS Research Institute, University of California, San Fransciso.

117. Plichta SB, Weisman CS, Nathanson CA, Ensminger ME, Robinson JC. Partner-specific condom use among adolescent women clients of a family planning clinic. *Journal of Adolescent Health Care*. 1992;13:506-511.

118. Pulerwitz J, Amaro H, De Jong W, Gortmaker SL, Rudd R. Relationship power, condom use and HIV risk among women in the USA. *AIDS Care*. 2002;14:789-800.

119. Wingood GM, DiClemente RJ. Childhood sexual abuse, HIV sexual risk and gender relations of African American women. *American Journal of Preventive Medicine*. 1997;13:380-384.

120. Raj A, Silverman JG, Amaro H. Abused women report greater male partner risk and gender-based risk for HIV: Finding from a community-based study with Hispanic women. *AIDS Care*. 2004;16:519-529.

121. Amaro H, Raj A. On the margin: Power and women's HIV risk reduction strategies. *Sex Roles*. 2000;42:723-749.

122. Heise L, Ellsberg M, Gottmoeller M. Ending violence against women. *Population Reports*. 1999;27:1-43.

123. Teitelman AM, Ratcliffe SJ, Morales-Aleman MM, Sullivan CM. Sexual relationship power, intimate partner violence, and condom use among minority urban girls. *Journal of Interpersonal Violence*. 2008;23:1694-1712.

124. Gutierrez LM, Oh HJ, Gillmore MR. Toward an understanding of (Em)Power(Ment) for HIV/AIDS prevention with adolescent women. *Sex Roles*. 2000;42:681-611.

125. Lane SD, Rubinstein RA, Keefe RH. Structural violence and racial disparity in HIV transmission. *Journal of Health Care for the Poor and Underserved*. 2004;15:319 - 335.

126. Sharpe TT, Glassman M, Collins C. The use of epidemiologic and other data in selecting behavioral HIV prevention interventions for African-American women. *Women & Health*. 2007;46:145 - 166.

127. Ryan S, Franzetta K, Manlove J, Holcombe E. Adolescents' discussions about contraception or STDs with partners before first sex. *Perspectives on Sexual and Reproductive Health*. 2007;39:149 - 157.

128. Dancy B, Berbaum. Condom use predictors for low-income African American women. *Western Journal of Nursing Research*. 2005;27:28-44.

129. Winningham A, Corwin S, Moore C, Richter D, Sargent R, Gore-Felton C. The changing age of HIV: sexual risk among older African American women living in rural communities. *Preventive Medicine*. 2004;39:809-814.

130. Widman L, Welsh D, McNulty J, Little K. Sexual communication and contraceptive use in adolescent dating couples. *Journal of Adolescent Health*. 2006;39:893-899.

131. Way N. Can't you see the courage, the strength that I have?: Listening to urban adolescent girls speak about their relationships. *Psychology of Women Quarterly*. 1995;19:107-128.

132. Biglan A, Metzler CW, Wirt R et al. Social and behavioral factors associated with high-risk sexual behaviors among adolescents. *Journal of Behavioral Medicine*. 1990;13:245-261.

133. Whitaker DJ, Miller KS, May DC, Levin ML. Teenage partners' communication about sexual risk and condom use: The importance of parent-teenager discussions. *Family Planning Perspectives*. 1999;31:117-121.

134. DiClemente RJ. Predictors of HIV-preventive sexual behavior in a high-risk adolescent population: The influence of perceived peer norms and sexual communication on incarcerated adolescents' consistent use of condoms. *Journal of Adolescent Health*. 1991;12:385-390.

135. Catania JA, Dolcini MM, Coates TJ et al. Predictors of condom use and multiple partnered sex among sexually-active adolescent women: Implications for AIDS-related health interventions. *Journal of Sex Research*. 1989;26:514-524.

136. Rickman RL, Lodico M, DiClemente RJ, Morris R, Baker C, Huscroft S. Sexual communication is associated with condom use by sexually active incarcerated adolescents. *Journal of Adolescent Health*. 1994;15:383-388.

137. Shoop DM, Davidson PM. AIDS and adolescents: The relation of parent and partner communication to adolescent condom use. *Journal of Adolescence*. 1994;17:137-148.

138. Brooks-Gunn J, Paikoff RL. "Sex is a gamble, kissing is a game": Adolescent sexuality and health promotion. In: Millstein SG, Petersen AC, Nightingale EO, eds. *Promoting the health of adolescents: New directions for the twenty-first century*. New-York: Oxford University Press; 1993:180-208.

139. Hofferth SL, Hayes CD. *Risking the Future: Adolescent Sexuality, Pregnancy, and Childbearing*. 2 vol. Washington, D.C.: National Academy Press; 1987.

140. Cvetkovitch G, Grote B. Psychological development and the social problem of teenage illegitimacy. In: Chilman C, ed. *Adolescent Pregnancy and Childbearing: Findings from Research*. Washington, DC: US Department of Health and Human Services; 1980:15-41.

141. Doljanac RF, Zimmerman MA. Psychological factors and high-risk sexual behavior: Race differences among urban adolescents. *Journal of Behavioral Medicine*. 1998;21:451-467.

142. Rodgers JL, Rowe DC. Social contagion and adolescent sexual behavior: A developmental and EMOSA model. *Psychological Review*. 1993;100:479-510.

143. Whitaker DJ, Miller KS. Parent-adolescent discussions about sex and condoms: Impact on peer influences of sexual risk behavior. *Journal of Adolescent Research*. 2000;15:251-273.

144. Corsaro WA, Eder D. Children's peer cultures. *Annual Review of Sociology*. 1990;16:197-220.

145. Brown LK, DiClemente RJ, Park T. Predictors of condom use in sexually active adolescents. *Journal of Adolescent Health*. 1992;13:651-657.

146. Fisher JD, Misovich SJ, Fisher WA. The impact of perceived social norms on adolescents' AIDS-risk behavior and prevention. In: DiClemente RJ, ed. *Adolescents and AIDS: A Generation in Jeopardy*. Beverly Hills, CA: Sage; 1992:117-136.

147. Costanzo PR, Shaw ME. Conformity as a function of age level. *Child Development*. 1966;37:967-975.

148. Handelsman CD, Cabral RJ, Weisfeld GE. Sources of information and adolescent sexual knowledge and behavior. *Journal of Adolescent Research*. 1987;2:455-463.

149. DiIorio C, Kelley M, Hockenberry-Eaton M. Communication about sexual issues: Mothers, fathers, and friends. *Journal of Adolescent Health*. 1999;24:181-189.

150. Emerson E, Donenberg G, Kapungu C. Attachment to mother or peers: What is protective for African American girls in psychiatric care? *Presented at the NIMH Annual Conference on the Role of Families in Preventing and Adapting to HIV/AIDS*. New York, NY; 2005.

151. Chodorow. Family structure and feminine personality. In: Rosaldo MZ, Lamphen L, eds. *Women, Culture, and Society*. Stanford, CA: Stanford University Press; 1974:43-66.

152. Chodorow N. Mothering, object-relations, and the female oedipal configuration. *Feminist Studies*. 1978;4:137-158.

153. Stevens JW. *Smart and Sassy: The Strengths of Inner-City Black Girls*. New York: Oxford University Press; 2002.

154. Surrey JL. The self-in-relation: A theory of women's development. In: Jordan JV, Kaplan AG, Miller JB, Stiver IP, Surrey JL, eds. *Women's Growth in Connection*. New York: Guilford; 1983:51-66.

155. Miller. B.C and Fox GL. Theories of adolescent heterosexual behavior. *Journal of Adolescent Research,*. 1987;2:269-282.

156. Miller BC, Benson B, Galbraith KA. Family relationships and adolescent pregnancy risk: A research synthesis. *Developmental Review*. 2001;21:1-38.

157. Hutchinson MK, Jemmott JB, III, Jemmott LS, Braverman P, Fong GT. The role of mother-daughter sexual risk communication in reducing sexual risk behaviors among urban adolescent females: A prospective study. *Journal of Adolescent Health*. 2003;33:98-107.

158. Hindelang RL, Dwyer WO, Leeming FC. Adolescent risk-taking behavior: A review of the role of parental involvement. *Current Problems in Pediatrics*. 2001;31:67-83.

159. Chewning B, Van Koningsveld R. Predicting adolescents' initiation of intercourse and contraceptive use. *Journal of Applied Social Psychology*. 1998;28:1245-1285.

160. Danziger SK. Family life and teenage pregnancy in the inner-city: Experiences of African American youth. *Children and Youth Services Review*. 1995;17:183-202.

161. Jaccard J, Dittus PJ, Gordon VV. Parent-adolescent congruency in reports of adolescents sexual behavior and in communications about sexual behavior. *Child Development*. 1998;69:247-261.

162. Longmore MA, Manning WD, Giordano PC. Preadolescent parenting strategies and teens' dating and sexual initiation: A longitudinal analysis. *Journal of Marriage and Family*. 2001;63:322-335.

163. Meschke LL, Bartholomae S, Zentall SR. Adolescent sexuality and parent-adolescent processes: Promoting healthy teen choices. *Journal of Adolescent Health*. 2002;31:264-279.

164. Resnick MD, Bearman PS, Blum RW et al. Protecting adolescents from harm: Findings from the National Longitudinal Study on Adolescent Health. *Journal of the American Medical Association*. 1997;278:823-832.

165. Upchurch D, Aneshensel C, Sucoff C, Levy-Storms L. Neighborhood and family contexts of adolescent sexual activity. *Journal of Marriage and the Family*. 1999;61:920-933.

166. Jones JB, Philliber S. Sexually active but not pregnant: A comparison of teens who risk and teens who plan. *Journal of Youth and Adolescence*. 1983;12:235-251.

167. Aronowitz T, Rennells RE, Todd E. Heterosocial behaviors in early adolescent African American girls: The role of mother-daughter relationships. *Journal of Family Nursing*. 2005;11:122-139.

168. O'Donnell L, Stueve A, Durnan R et al. Parenting practices, parents' underestimation of daughters' risks, and alcohol and sexual behaviors of urban girls *Journal of Adolescent Health*. 2008;42:496-502.

169. Inazu JK, Fox GL. Maternal influence on the sexual behavior of teen-age daughters. *Journal of Family Issues*. 1980;1:81-102.

170. McKay MM, Baptiste D, Coleman D et al. Preventing HIV risk exposure in urban communities: The CHAMP family program. In: Pequegnat W, Szapocznik J, eds. *Working with Families in the Era of HIV/AIDS*. London: Sage Publications, Inc; 2000:67-87.

171. Stauffer A, Emerson E, Donenberg G. Psychopathology and adolescent sexual risk taking: The role of maternal attachment. *Presented at the NIMH Annual Conference on the Role of Families in Preventing and Adapting to HIV/AIDS*. San Juan, Puerto Rico; 2006.

172. Metzler CW, Noell J, Biglan A, Ary D, Smolkowski K. The social context for risky sexual behavior among adolescents. *Journal of Behavioral Medicine*. 1994;17:419-438.

173. Li X, Feigelman S, Stanton B. Perceived parental monitoring and health risk behaviors among urban low-income African-American children and adolescents. *Journal of Adolescent Health*. 2000;27:43-48.

174. Miller KS, Levin ML, Whitaker DJ, Xu X. Patterns of condom use among adolescents: The impact of mother-adolescent communication. *American Journal of Public Health*. 1998;88:1542-1544.

175. Romer D, Black M, Ricardo I et al. Social influences on the sexual behavior of youth at risk for HIV exposure. *American Journal of Public Health*. 1994;84:977-985.

176. Romer D, Stanton B, Galbraith J, Feigelman S, Black MM, Li X. Parental influence on adolescent sexual behavior in high-poverty settings. *Archives of Pediatric and Adolescent Medicine*. 1999;153:1055-1062.

177. DiClemente RJ, Wingood GM, Crosby R et al. Parental monitoring: Association with adolescents' risk behaviors. *Pediatrics*. 2001;107:1363-1368.

178. Davis EC, Friel LV. Adolescent sexuality: Disentangling the effects of family structure and family context. *Journal of Marriage and Family*. 2001;63:669-681.

179. Mounts NS. Young adolescents' perceptions of parental management of peer relationships. *Journal of Early Adolescence*. 2001;21:92-122.

180. Werner NE, Silbereisen RK. Family relationship quality and contact with deviant peers as predictors of adolescent problem behaviors: The moderating role of gender. *Journal of Adolescent Research*. 2003;18:454-480.

181. Wilson H, Emerson E, Donenberg G. Risky sexual behavior in clinically disturbed girls: Which relationships matter - parents, peers, or partners? *Annual NIMH Conference on the Role of Families in Preventing and Adapting to HIV/AIDS*. Los Angeles, CA; 2001.

182. Buckner JC, Mezzacappa E, Beardslee WR. Characteristics of resilient youths living in poverty: The role of self-regulatory processes. *Development and Psychopathology*. 2003;15:139-162.

183. Jarret RL. African American family and parenting strategies in impoverished neighborhoods. *Qualitative Sociology*. 1997;20:275-288.

184. McBride Murray V. Variation in adolescent pregnancy status: A national tri-ethnic study. In: McCubbin HI, Thompson EA, Thompson AI, Futrell JA, eds. *Resiliency in African American Families*. Thousand Oaks, CA: Sage Publications; 1998:179-206.

185. Kotchick BA, Forehand R. Putting parenting in perspective: A discussion of the contextual factors that shape parenting practices. *Journal of Child and Family Studies*. 2002;11:255-269.

186. Miller KS, Forehand R, Kotchick BA. Adolescent sexual behavior in two ethnic minority samples: The role of family variables. *Journal of Marriage and the Family*. 1999;61:85-98.

187. Dutra R, Miller KS, Forehand R. The process and content of sexual communication with adolescents in two-parent families: Associations with sexual risk-taking behavior. *AIDS and Behavior*. 1999;3:59-66.

188. Kastner LS. Ecological factors predicting adolescent contraceptive use: Implications for intervention. *Journal of Adolescent Health Care*. 1984;5:79-86.

189. East PL. The younger sisters of childbearing adolescents: Their attitudes, expectations, and behaviors. *Child Development*. 1996;67:267-282.

190. Miller BC, Norton MC, Fan X, Christopherson CR. Pubertal development, parental communication, and sexual values in relation to adolescent sexual behaviors. *Journal of Early Adolescence*. 1998;18:27-52.

191. Moore KA, Peterson JL, Furstenberg FF. Parental attitudes and the occurrence of early sexual activity. *Journal of Marriage and the Family*. 1986;48:777-782.

192. Fisher TD. Family communication and the sexual behavior and attitudes of college students. *Journal of Youth and Adolescence*. 1987;16:481-495.

193. Lock SE, Vincent ML. Sexual decision-making among rural adolescent females. *Health Values: The Journal of Health Behavior, Education, & Promotion*. 1995;19:47-58.

194. Miller K, Fasula A, Dittus P, Wiegand R, Wyckoff S, McNair L. Barriers and facilitators to maternal communication with preadolescents about age-relevant sexual topics. *AIDS and Behavior*. 2007.

195. Werner-Wilson RJ. Are virgins at risk for contracting HIV/AIDS? *Journal of HIV/AIDS Prevention & Education for Adolescents & Children*. 1998;2:63-71.

196. Bynum MS. African American mother-daughter communication about sex and daughters' sexual behavior: Does college racial composition make a difference? *Cultural Diversity and Ethnic Minority Psychology*. 2007;13:151-160.

197. Moore MR, Chase-Lansdale PL. Sexual intercourse and pregnancy among African American girls in high-poverty neighborhoods: The role of family and perceived community environment. *Journal of Marriage and Family*. 2001;63:1146-1157.

198. DiIorio C, McCarty F, Denzmore P, Landis A. The moderating influence of mother-adolescent discussion on early and middle African American adolescent sexual behavior. *Research in Nursing and Health*. 2007;30:193 - 202.

199. Meneses L, Orrell-Valente J, Guendelman S, Oman D, Irwin C. Racial/ethnic differences in mother-daughter communication about sex. *Journal of Adolescent Health*. 2006;39:128-131.

200. Nappi C, McBride C, Donenberg G. HIV/AIDS communication among adolescents in psychiatric care and their parents. *Journal of Family Psychology*. 2007;21:637-644.

201. Hadley W, Brown LK, Lescano CM et al. Parent-adolescent sexual communication: Associations of condom use with condom discussions. *AIDS and Behavior*. 2009;13:997-1004.

202. Crosby RA, DiClemente RJ, Wingood GM, Cobb BK, Harrington K, Davies SL. HIV/STD-protective benefits of living with mothers in perceived supportive families: A study of high-risk African American female teens. *Preventive Medicine*. 2001;33:175-178.

203. Ramirez-Valles J, Zimmerman MA, Juarez L. Gender differences of neighborhood and social control processes: A study of the timing of first intercourse among low-achieving, urban, African American youth. *Youth & Society*. 2002;33:418-441.

204. Cauce AM, Hiraga Y, Graves D, Gonzalez N, Ryan-Finn K, Grove K. African American mothers and their adolescent daughers: Closeness, conflict, and control. In: Leadbeater BJR, Way N, eds. *Urban Girls: Resisting Stereotypes, Creating Identities*. New York: NYU Press; 1996:100-116.

205. Feldman S, Rosenthal D. The effect of communication characteristics on family members' perceptions of parents as sex educators. *Journal of Research on Adolescence*. 2000;10:119-150.

206. Fox G. The family's role in adolescent sexual behavior. In: Ooms T, ed. *Teenage Pregnancy in a Family Context*. Philadelphia, PA: Temple University Press; 1981:73-130.

207. Hepburn EH. A three-level model of parent-daughter communication about sexual topics. *Adolescence*. 1983;18:523-534.

208. Kahn JR, Smith, K.W., Roberts, E.J. *Family Communication and Adolescent Sexual Behavior*. Cambridge, MA: American Institute for Research; 1984.

209. Miller KS, Kotchick BA, Dorsey S, Forehand R, Ham AY. Family communication about sex: What are parents saying and are their adolescents listening? *Family Planning Perspectives*. 1998;30:218-222 & 235.

210. Rozema HJ. Defensive communication climate as a barrier to sex education in the home. *Family Relations: Journal of Applied Family and Child Studies*. 1986;35:531-537.

211. Grant K, Poindexter, L., et al. Economic stress and psychological distress among urban African American adolescents: The mediating role of parents. *Journal of Prevention and Intervention in the Community*. 2000;20:25-36.

212. Miller KS, Forehand R, Kotchick BA. Adolescent sexual behavior in two ethnic minority groups: A multi-system perspective. *Adolescence*. 2000;35:313-333.

213. Repetti RL, Taylor, S.E., Seeman, T.E. Family social environmnets and the mental and physical health of offspring. *Psychological Bulletin*. 2002;128:330-366.

214. Kotchick BA, Shaffer A, Miller KS, Forehand R. Adolescent sexual risk behavior: A multi-system perspective. *Clinical Psychology Review*. 2001;21:493-519.

215. Somers CL, Paulson SE. Students' perceptions of parent-adolescent closeness and communication about sexuality: Relations with sexual knowledge, attitudes, and behaviors. *Journal of Adolescence*. 2000;23:629-644.

216. Dittus P, Jaccard J, Gordon VV. Direct and nondirect communication of maternal beliefs to adolescents: Adolescent motivations for premarital sexual activity. *Journal of Applied Social Psychology,*. 1999;29:1927-1963.

217. Kotchick BA, Dorsey S, Miller KS, Forehand R. Adolescent sexual risk-taking behavior in single-parent ethnic minority families. *Journal of Family Psychology*. 1999;13:93-102.

218. Mott FL, Fondell, M.M., Hu, P.N., Kowalewski-Jones, L., and Menaghan, E. G. The determinants of first sex by age 14 in a high-risk adolescent population. *Family Planning Perspectives*. 1996;28:13-18.

219. Newcomer SF, Udry JR. Parent-child communication and adolescent sexual behavior. *Family Planning Perspectives*. 1985;17:169-174.

220. Serbin LA, Cooperman JM, Peters PL, Lehoux PM, Stack DM, Schwartzman AE. Intergenerational transfer of psychosocial risk in women with childhood histories of aggression, withdrawal, or aggression and withdrawal. *Developmental Psychology*. 1998;34:1246-1262.

221. Hogan D, Kitagawa E. The impact of social status, family structure, and neighborhood on the fertility of black adolescents. *American Journal of Sociology*. 1985;90:825-855.

222. Coatsworth JD, Pantin H, McBride C, Briones E, Kurtines W, Szapocznik J. Ecodevelopmental correlates of behavior problems in young Hispanic families. *Applied Developmental Science*. 2000;6:126 - 143.

223. Henrich C, Brookmeyer K, Shrier L, Shahar G. Supportive relationships and sexual risk behavior in adolescence: An Ecological-Transactional approach. *Journal of Pediatric Psychology*. 2006;31:286-297.

224. Collins WA, Hennighausen KC, Schmit DT, Sroufe AL. Developmental precursors of romantic relationships: A longitudinal analysis. In: Shulman S, Collins WA, eds. *Romantic Relationships in Adolescence: Developmental Perspectives*. 78 vol. San Francisco: Jossey-Bass; 1997:69-84.

225. Bowlby J. Attachment and loss: Separation. In: Basic Books I, ed. *Separation*. 2 vol. New York; 1973:201-257.

226. Sroufe A, Fleeson J. Attachment and the construction of relationships. In: Hartup WW, Rubin Z, eds. *Relationships and development*. New Jersey: Lawrence Erlbaum; 1986.

227. Hoffman M. Moral internalization, parental power, and the nature of parent-child interaction. *Developmental Psychology*. 1975;11:228 - 239.

228. Hutchinson MK, Cooney TM. Patterns of parent-teen sexual risk communication: Implications for intervention. *Family Relations*. 1998;47:185-194.

229. DiClemente RJ, Wingood GM, Crosby R, Cobb BK, Harrington K, Davies SL. Parent-adolescent communication and sexual risk behaviors among African American adolescent females. *The Journal of Pediatrics*. 2001;139:407-412.

230. Fox GL, Inazu JK. Patterns and outcomes of mother-daughter communication about sexuality. *Journal of Social Issues*. 1980;36:7-29.

231. Dembo R, Belenko S, Childrs K, Wareham J, Schmeidler J. Individual and community risk factors and sexually transmitted diseases among arrested youths: A two level analysis. *Journal of Behavioral Medicine*. 2009.

232. Shaw CR, McKay HD. Juvenile delinquency and urban areas. *University of Chicago Press*. 1969.

233. Sampson RJ, Groves WB. Community structures and crime: testing social disorganization theory. *American Journal of Sociology*. 1989;94:774-802.

234. Browning CR, Leventhal T, Brooks-Gunn J. Sexual initiation in early adolescence: the nexus of parental and community control. *American Sociological Review*. 2005;70:758-778.

235. Weibush R, Freitag R, Baird C. Preventing delinquency through improved child protection services. *Juvenile Justice Bulletin*. 2001.

236. Rodgers J, Rowe DC. Influence of Siblings on Adolescent Sexual Behavior. *Developmental Psychology*. 1988;24:722-728.

237. Schiff M, El-Bassel N, Engstrom M, Gilbert L. Psychological distress and intimate sexual abuse among women in methadone maintanence treatment program. *Social Science Review*. 2002;76:302-320.

238. Liebschutz JM, Feinman G, Sullivan L, Stein M, Samet J. Physical and sexual abuse in women infected with the human immunodeficiency virus: Increased illness and health care organization. *Archives of Internal Medicine*. 2000;160:1659-1664.

239. Donenberg GR, Pao M. Understanding HIV/AIDS: Psychosocial and psychiatric issues in youths. *Contemporary Psychiatry*. 2003;2:1-8.

240. Sagrestano LM, McCormick SH, Paikoff RL, Holmbeck GN. Pubertal development and parent-child conflict in low-income, urban, African American adolescents. *Journal of Research on Adolescence*. 1999;9:85-107.

241. Miller BC, Moore KA. Adolescent sexual behavior, pregnancy, and parenting: Research through the 1980's. *Journal of Marriage and the Family*. 1990;52:1025-1044.

242. Gabel S, Stallings, Michael, et al. Personality dimensions and substance misuse: Relationships in adolescent mothers and fathers. *American Journal on Addictions,*. 1999;8:101-113.

243. Hops H, Duncan TE, Duncan SC, Stoolmiller M. Parent substance use as a predictor of adolescent use: A six-year lagged analysis. *Annals of Behavioral Medicine*. 1996;18:157-164.

244. Tarter R, Schultz K, Kirisci L, Dunn M. Does living with a substance abusing father increase substance abuse risk in male offspring? Impact on individual, family, school, and peer vulnerability factors. *Journal of Child and Adoloscent Substance Abuse.* 2001;10:59-70.

245. Sherman SG, Gielen AC, McDonnell KA. Power and attitudes in relationships (PAIR) among a sample low-income, African-American women: Implications for HIV/AIDS prevention. *Sex Roles*. 2000;42:283-294.

246. Centers for Disease Control and Prevention. HIV/AIDS Prevention Research Synthesis Project: Compendium of HIV Prevention Interventions with Evidence of Effectiveness. Atlanta; 1999.

247. Dilorio C, Resnicow K, McCarty F et al. Keepin' It R.E.A.L.! Results of a mother-adolescent HIV prevention program. *Nursing Research*. 2006;55:43-51.

248. Wu Y, Stanton B, Galbraith J et al. Sustaining and broadening intervention impact: A longitudinal randomized trial of 3 adolescent risk reduction approaches. *Pediatrics*. 2003;111:32-38.

249. McKay MM, Chasse KT, Paikoff R et al. Family-level impact of the CHAMP family program: A community collaborative effort to support urban families and reduce youth HIV risk exposure. *Family Process*. 2004;43:79-93.

250. Krauss BJ, et al. Saving our children from a silent epidemic: The PATH program for parents and preadolescents. In: Pequegnat WaS, J., ed. *Working with Families in the Era of HIV/AIDS*. Thousand Oaks: SAGE Publications, Inc.; 2000:89-112.

251. Forehand R, Armistead L, Long N et al. Efficacy of a parent-based sexual-risk prevention program for African American preadolescents. *ARchives of Pediatric Adolescent Medicine*. 2007;161:1123-1129.

252. Dancy B, Crittenden K, Talashek M. Mothers' effectiveness as HIV risk reduction educators for adolescent daughters. *Journal of Health Care for the Poor ad Underserved*. 2006;17:218-239.

253. DiClemente RJ, Wingood GM, Harrington KF et al. Efficacy of an HIV prevention intervention for African American adolescent girls: A randomized controlled trial. *Journal of the American Medical Association*. 2004;292:171-179.

254. Centers for Disease Control and Prevention. Updated Compendium of Evidenced-Based Interventions; 2007.

255. Wingood GM, DiClemente RJ. Enhancing adoption of evidence-based HIV interventions: Promotion of a suite of HIV prevention interventions for African American women. *AIDS Education and Prevention*. 2006;18:161-170.

256. Collins CE, Whiters DL, Braithwaite R. The Saved SISTA Project: A faith-based HIV prevention program for black woemn in addiction recovery. *American Journal of Health Studies*. 2007;22:76-82.

257. Cornelius J, Moneyham L, LeGrand S. Adaptation of an HIV prevention curriculum for use with older African American women. *Journal of the Association of Nurses in AIDS Care*. 2008;19:16 - 27.

258. DiClemente R. Development and Evaluation of an HIV Risk-Reduction Intervention Tailored for High-Risk African-American Female Adolescents Seeking Treatment at STD Clinics. *Archives of Pediatrics and Adolescent Medicine*. in press.

259. Odgers CL, Caspi A, Broadbent JM. Prediction of differential adult health burden by conduct problem subtypes in males. *Archives of General Psychiatry*. 2007;64:476-484.

260. Petras H, Kellam SG, Brown C, et al. Developmental epidemiological courses leading to antisocial personality disorder and violent and criminal behavior: Effects by young adulthood of a universal preventive intervention in ﬁrst- and second-grade classrooms. *Drug and Alcohol Dependence*. 2008;95:S45-59.

261. Centers for Disease Control and Prevention. Youth Risk Behavior Survey 1999: U.S. Department of Health and Human Services; 2000.

262. Smith C. Factors associated with early sexual activity among urban adolescents. *Social Work*. 1997;42:334-346.

263. Parfenoff SH, McCormick A. Parenting preadolescents at risk: Knowledge, attitudes, and communication about HIV/AIDS. *the annual meeting for the Society for Research in Child Development*. Washington, D.C.; 1997.

264. Madison SM, McKay, M.M., Paikoff, R.L., and Bell, C. Basic research and community collaboration: Necessary ingredients for the development of a family-based HIV prevention program. *AIDS Education and Prevention*. 2000;12:281-298.

265. McKay M, Baptiste D, McCormick A, Scott R, Paikoff RL. Family based HIV risk exposure prevention programming: A description of the CHAMP family intervention. *at the annual meeting at the International Conference on AIDS*. Vancouver, BC; 1996.

266. Weisz JR. Personal Communication; 1998.

267. Paikoff RL, Parfenoff SH, Holmbeck GN, Bhorade AM, Gillming G. Family, friendship, and social problem solving factors in exposure to sexual possibility situations among pre-adolescent urban African-American youth. in preparation.

268. Stouthamer-Loeber M, Van Kammen W. *Data Collection and Management: A Practical Guide*. Thousand Oaks, CA: Sage Publications; 1995.

269. Bellg AJ, Borrelli B, Resnick B et al. Enhancing treatment fidelity in health behavior change studies: Best practices and recommendations from the NIH Behavior Change Consortium. *Health Psychology*. 2004;23:443-451.

270. Jemmott JBI, Jemmott LS, Fong GT. Reductions in HIV risk-associated sexual behaviors among Black male adolescents: Effects of an AIDS prevention intervention. *American Journal of Public Health*. 1992;82:372-377.

271. Di Noia J, Schinke SP, Pena JB, Schwinn TM. Evaluation of a brief computer-mediated intervention to reduce HIV risk among early adolescent females. *Journal of Adolescent Health*. 2004;35:62-64.

272. Wingood GM, Card JJ, DiClemente RJ. Efficacy of a computer-based HIV Intervention for African American Women. *Journal of Health Psychology*. under review.

273. Belcher L, Kalichman S, Topping M et al. A randomized trial of a brief HIV risk reduction counseling intervention for women. *Journal of Consulting and Clinical Psychology*. 1998;5:856-861.

274. Bolu OO, Lindsey C, Kamb ML et al. Is HIV/sexually transmitted disease prevention counseling effective among vulnerable populations? A subset analysis based on data collected for a randomized controlled trial evaluating counseling efficacy (Project RESPECT). *Sexually Transmitted Diseases*. 2004;31:469-474.

275. Chernoff RA, Davison GC. An evaluation of a brief HIV/AIDS prevention intervention for college students using normative feedback and goal setting. *AIDS Education and Prevention*. 2005;17:91-104.

276. Jemmott LS, Jemmott JB, O'Leary A. Effects on sexual risk behavior and STD rate of brief HIV/STD prevention interventions for African American Women in primary care settings: Effects on sexual risk behavior and STD incidence. *American Journal of Public Health* 2007;97:1034-1040.

277. Jemmott LS, Jemmott JB, Hutchinson MK, Cederbaum J, O'Leary A. Translating research into practice: STI/HIV risk-reduction interventions in clinical practice settings. *JOGNN*. 2008;37:137-145.

278. Kamb ML, Fishbein M, Douglas JM et al. Efficacy of risk-reduction counseling to prevent human immunodeficiency virus and sexually transmitted diseases: a randomized controlled trial. Project RESPECT Study Group. *Journal of the American Medical Association*. 1998;13:1161-1167.

279. Jemmott JB, III, Jemmott LS, Fong GT. Abstinence and safer sex HIV risk-reduction interventions for African American adolescents: A randomized controlled trial. *Journal of the American Medical Association*. 1998;279:1529-1536.

280. Jemmott JB, III, Jemmott LS, Fong GT, McCaffree K. Reducing HIV risk-associated sexual behavior among African American adolescents: Testing the generality of intervention effects. *American Journal of Community Psychology*. 1999;27:161-187.

281. Jemmott JB, III, Jemmott LS, Braverman PK, Fong GT. HIV/STD risk reduction interventions for African American and Latino adolescent girls at an adolescent medicine clinic: A randomized controlled trial. *Archives of Pediatric and Adolescent Medicine*. 2005;159:440-449.

282. Donenberg G, Brown LK, Lescano C, Hadley W, Kapungu C. Project STYLE: A multi-site family-based HIV prevention program for adolescents with psychiatric disorders. In: Pequegnat W, Bell C, eds. *Families and HIV/AIDS*. New York: Springer; in press.

283. Boekeloo BO, Schiavo L, Rabin DL, Conlon RT, Jordan CS, Mundt DJ. Self-reports of HIV risk factors by patients at a sexually transmitted disease clinic: Audio vs written questionnaires. *American Journal of Public Health*. 1994;84:754-760.

284. Romer D, Hornik R, Stanton B et al. "Talking" computers: A reliable and private method to conduct interviews on sensitive topics with children. *The Journal of Sex Research*. 1997;34:3-9.

285. T. Achenbach T, McConaughy SH, Howell CT. Child/adolescent behavioral and emotional problems: Implications of cross-informant correlations for situational specificity. *Psychological Bulletin*. 1987;101:213-232.

286. Paikoff R. Personal Communication; 1998.

287. Fisher JD, Misovich, S.J. "Personal Communication"; 1998.

288. DiClemente RJ. HIV and AIDS Knowledge Questionnaire. Birmingham, AL: University of Alabama Departments of Medicine and Pediatrics; 1994.

289. Misovich SJ, Fisher WA, Fisher JD. A measure of AIDS prevention, information, motivation, behavioral skills, and behavior. In: Davis C, Yarber W, Bauserman R, Schreer G, Davis S, eds. *Handbook of Sexuality-Related Measures*. Thousand Oaks, CA: Sage; 1998:328-337.

290. Boekhout BA, Hendrick SS, Hendrick C. Exploring infidelity: Developing the relationship issues scale. *Journal of Loss and Trauma*. 2003;8:283-306.

291. Achenbach T. *Manual for the Child Behavior Checklist/ 4-18 and 1991 Profile*. Burlington, VT: University of Vermont, Department of Psychiatry; 1991.

292. Achenbach T. *Manual for the Youth Self-Report and 1991 Profile*: Department of Psychiatry University of Vermont; 1991.

293. Parker JDA, Taylor GJ, Bagby RM. The 20-item Tronto alexithymia scale III. Reliability and factorial validity in a community population. *Journal of Psychosomatic research*. 2003;55:269-275.

294. Derogatis LR. *SCL-90-R: Administration, scoring and procedures manual*. Townsend: Clinical Psychometric Research, Inc.; 1992.

295. Sellers RM, Morgan L, Brown TN. A multidimensional approach to racial identity: Implications for African American children. In: Neal-Barnett A, ed. *Forging links: Clinical-developmental perspectives on African American children*. West Port, CT: Praeger; 2001:23-56.

296. Resnicow K, Soler R, Braithwaite RL, Ben Selassie M, Smith M. Development of a racial and ethnic identity scale for African American adolescents: The survey of black life. *Journal of Black Psychology*. 1999;25:171-188.

297. El-Bassel N, Ivanoff A, Schilling RF, Gilbert L, Bourne D, Chen D-R. Preventing HIV/AIDS in drug-abusing incarcerated women through skills-building and social support enhancement: Preliminary outcomes. *Social Work Research*. 1995;19:131-141.

298. Jessor R, Jessor SL. *Problem Behavior and Psychosocial Development: A Longitudinal Study of Youth*. New York: Academic; 1977.

299. Costa FM, Jessor R, Fortenberry JD, Donovan JE. Psychosocial conventionality, health orientation, and contraceptive use in adolescence. *Journal of Adolescent Health*. 1996;18:404-416.

300. Costa FM, Jessor R, Donovan JE, Fortenberry JD. Early initiation of sexual intercourse: The influence of psychosocial unconventionality. *Journal of Research on Adolescence*. 1995;5:93-121.

301. Donovan JE, Jessor R, Costa FM. Adolescent health behavior and conventionality-unconventionality: An extension of problem-behavior theory. *Health Psychology*. 1991;10:52-61.

302. Armsden GC, Greenberg MT. The inventory of parent and peer attachment: Individual differences and their relationship to psychological well-being in adolescence. *Journal of Youth and Adolescence*. 1987;16:427-454.

303. Greenberg MT. Personal Communication; 2002.

304. Oregon Social Learning Center. Parental Monitoring and Supervision Constructs. (Technical reports). Eugene, Oregon: Oregon Social Learning Center; 1990.

305. NIDA. *Prevalence of Drug Use in the DC Metropolitan Area Adult and Juvenile Offender Populations: 1991*. Rockville, MD: USDHHS; 1995.

306. IBS. Denver Youth Survey Youth Interview Schedule. Boulder, CO: University of Colorado; 1991.

307. Dowling S, Johnson ME, Fisher DG. Reliability of drug users' self-report of recent drug use. *Assessment*. 1994;1:382-392.

308. Needle R, Fisher DG, Weatherby N et al. Reliability of self-reported HIV risk behaviors of drug users. *Psychology Addicted Behavior*. 1995;9:242-250.

309. Weatherby NL, Needle R, Cesari H. Validity of self-reported drug use among injection drug users and crack cocaine users recruited through street outreach. *Education Program Plan*. 1994;17:347-355.

310. Watters JK. Street Youth at Risk for AIDS. Rockville, MD: National Institute on Drug Abuse; 1994.

311. Carroll KC, Aldeen WE, Morrison M, Anderson R, Lee D, Mottice S. Evaluation of the abbott LCx ligase chain reaction assay for detection of chlamydia trachomatis and neisseria gonorrhoeae in urine and genital swab specimens from a sexually transmitted disease clinic population. *Journal of Clinical Microbiology*. 1998;36:1630-1633.

312. Gaydos C, Howell, M., et al. Use of ligase chain reaction with urine versus cervical culture for detection of Chlamydia trachomatis in an asymptomatic military population of pregnant and nonpregnant females attending Papnicolaou smear clinics. *Journal of Clinical Microbiology,*. 1998;36:1300-1304.

313. Donenberg GR, Bryant FB, Emerson E, Wilson HW, Pasch KE. Tracing the roots of early sexual debut among adolescents in psychiatric care. *Journal of the American Academy of Child and Adolescent Psychiatry*. 2003;42:594-608.

314. Wilson H, Donenberg G. Quality of parent communication about sex and its relationship to mentally ill adolescents' risky sexual behavior. *Journal of Child Psychology and Psychiatry and Allied Disciplines*. 2004;45:387-395.

315. Hollingshead AB. Four factor index of social status. New Haven, CT.: Yale University; 1975.

316. Boyd A, Jordan K, Kach J, Ware Spencer N, Donenberg G. Does mother’s relationship status influence sexual behaviors among African-American girls. *Poster presented at the NIMH Annual Conference on the Role of Families in preventing and adapting to HIV/AIDS*. San Juan, Puerto Rico; 2006.

317. Emerson E, Donenberg G, Stauffer A, Mustanski B, Jordan K. The influence of African-American mothers’ sexual behavior and attitudes in sexual relationships on their teen daughters’ behavior and attitudes *Presented at the Society for the Scientific Study of Sexuality*. Las Vegas, Nevada; 2006.

318. Donenberg G. Adolescence. In: Loue S, Sajatovic M, eds. *Encyclopedia of Women’s Health*. New York: Kluwer Academic/Plenum Publishing; 2004.

319. Jordan K, Boyd A, Donenberg G. Sexual behavior and self-efficacy among African American mothers and daughters. *Presented at the NIMH Annual Conference on the Role of Families in Preventing and Adapting to HIV/AIDS*. Brooklyn, NY; 2005.

320. Jordan K, Boyd A, Lampe R, Donenberg G. Parent-Adolescent communication patterns among African Americans: Do mothers raise their daughters and love their sons? *Poster presented at the NIMH conference on the Role of Families in Preventing and Adapting to HIV/AIDS*. Brooklyn, NY; 2004.

321. Jordan K, Eisenberg J, Emerson E, Donenberg G. Comparing African-American mothers’ and daughters’ romantic relationship behavior: A qualitative study in a psychiatric setting. *Presented at the NIMH Annual Conference on the Role of Families in Preventing and Adapting to HIV/AIDS*. Washington, D.C.; 2003.

322. Robinson M. Effects of Family Factors, Relationship Schemas, Communication about Sex, and Sexual Behavior Progression on the Risky Sexual Behaviors of African American Adolescent Females. *Department of Clinical Psychology*. PhD vol. Chicago: Loyola University Chicago; 2007.

323. Kekwaletswe TC. Africentric values and ethnic identity as protective or risk mechanisms for risky sex, psychopathology and substance use in African American youth. *Department of Psychology, Finch University of Health Sciences/The Chicago Medical School.* Ph.D. vol; 2007.

324. Ware Spencer N. The Association of Religion and Sexual Behaviors among African American Adolescent Girls Seeking Psychiatric Care. *Department of Psychology*. PhD vol. Chicago: Argosy University; 2007.

325. Phinney JS. The multigroup ethnic identity measure: A new scale for use with diverse groups. *Journal of Adolescent Research*. 1992;7:156-176.

326. Belgrave FZ, Townsend TG, Cherry VR, Cunningham DM. The influence of an Africentric worldview and demographic variables on drug knowledge, attitudes, and use among African American youth. *Journal of Community Psychology*. 1997;25:421-433.

327. Folkman S, Lazarus RS. Stress processes and depressive symptomology. *Journal of Abnormal Psychology*. 1996;95:107-113.

328. Utsey S, Adams E, Bolden M. Development and Initial Validation of the Africultural Coping Systems Inventory *Journal of Black Psychology*. 2000;26:194-215.

329. Brown LK, Hadley W, Stewart A et al. Psychiatric disorders and sexual risk among adolescents in mental health treatment. *Journal of Consulting and Clinical Psychology*. in press.

330. Nappi C, Thakral C, Kapungu C et al. Parental monitoring as a moderator of the effect of family sexual communication on sexual risk behavior among adolescents in psychiatric care. *AIDS and Behavior*. 2009;13:1012-1020.

331. Wingood GM, DiClemente RJ. The ADAPT-ITT Model: A novel method of adapting evidence-based HIV interventions. *Journal of Acquired Immune Deficiency Syndrome*. 2008;47:S40-S46.

332. Little RJA, Rubin DB. *Statistical Analysis with Missing Data, 2nd Edition*. New York: Wiley; 2002.

333. Ma G, Troxel AB, Heitjan DF. An index of local sensitivity to nonignorable dropout in longitudinal modeling. *Statistics in Medicine*. 2005;24:2129-2150.

334. Xie H. A local sensitivity analysis approach to longitudinal non-gaussian data with nonignorable dropout. *Statistics in Medicine*. 2008;27:3155-3177.

335. Cohen J, Cohen P. *Applied multiple regression/correlation analysis for the behavioral sciences*. 2nd edition ed. New York: Erlbaum; 1983.

336. Baron RM, Kenny DA. The moderator-mediator variable distinction in social psychological research: Conceptual, strategic, and statistical considerations. *Journal of Personality and Social Psychology*. 1986;51:1173-1182.

337. AIDS Foundation of Chicago. Women and HIV/AIDS; 2009.

338. Illinois Department of Public Health. Illinois HIIV/AIDS/STD Monthly Surveillance Update; 2009.

339 A. Costello et al., Development and testing of the NIMH diagnostic interview

schedule for children in a clinic population: Final report (Rockville, MD: Center for Epidemiologic Studies, NIMH, 1984) Contract RFP-DB081-0027.

340 D. Shaffer et al., “Diagnostic Interview for Children (DISC 2.3) --Child Version,” Columbia University, 1991.

341 D. Shaffer et al., “Diagnostic Interview for Children (DISC 2.3) --Parent Version,” Columbia University, 1991.

342 M. Schwab-Stone et al., “Criterion validity of the NIMH Diagnostic Interview Schedule for Children,” Journal of the American Academy of Child and Adolescent Psychiatry 35 (1996): 878-888.

343 M. Schwab-Stone et al., “The Diagnostic Interview Schedule for Children-Revised Version (DISC-R): II. Test-retest reliability,” Journal of the American Academy of Child and Adolescent Psychiatry 32 (1993): 651-657.

344 D. Shaffer et al., “The diagnostic interview schedule for children - revised version (DISC-R): I. Preparation, field testing, interrater reliability, and acceptability,” Journal of the American Academy of Child and Adolescent Psychiatry 32 (1993): 643-650.

345 L. Lucas, “Personal Communication,” (1998).

346 J. R. Udry et al., The National Longitudinal Study of Adolescent Health, 1998, website, Carolina Population Center at the University of North Carolina at Chapel Hill, Available: http://www.cpc.unc.edu/addhealth/design.html2001.

347 Nadal, K. L. (2011). The Racial and Ethnic Microaggressions Scale (REMS): Construction, Reliability, and Validity. Journal of Counseling Psychology, 58, 470–480.

348 Connor-Smith, J. K., Compas, B. E., Wadsworth, M. E., Thomsen, A. H., & Saltzman, H. (2000). Responses to stress in adolescence: Measurement of coping and involuntary stress responses. Journal of Consulting and Clinical Psychology, 68, 976-992.

349 Ybarra, M. L., Korchmaros, J., Kiwanuka, J., Bangsberg, D. R., & Bull, S. (2012). Examining the applicability of the IMB model in predicting condom use among sexually active secondary school students in Mbarara, Uganda. AIDS & Behavior.

**IMARA Amendments**

**Amendment #1**, approved on 7/27/2011, was a request for a signature from the Vice Chancellor for Research for our certificate of confidentiality application.

**Amendment #2**, approved on 9/20/2011, added key personnel.

**Amendment #3,** approved on 3/15/2012, submitted 1) NIH approved COC letter, 2) added key personnel, updated recruitment sites with FWAs and permission letters, 3) updated protocol/initial application: changed the name of the study, clarified locations where research activities will occur, added another clinician to assist with risk assessment, opened the possibility of non-AA women to conduct the research interviews, updated incentive procedures, clarified data security procedures, deleted any mention of Facebook, and updated the PIs address, 4) updated the measures and intervention curriculums, 5) updated and clarified STI testing, treatment, and reporting procedures, 6) updated consent and recruitment documents to reflect Amendment 3 updates, and 7) updated the study's confidentiality agreement to include new project title.

**Amendment #4**, approved on 8/27/2012, did the following: 1) Revised IMARA curriculums, 2) edited Appendix P for information changes and added key personnel, 3) revised Program Flyer and Recruitment Letter, 4) revised Eligibility Screening Questionnaire, 5) revised protocol measures to a) add sexual self-agency items, b) moved mother-report sexual relationship power and partner sexual communication items to Mother ARBA, c) added, deleted or revised items to the following measures: Relationship Power Scale-Girl Report, ARBA-Mothers Report, ARBA-Girls Report, Adolescent Romantic Relationship Scale and Boyfriend/Girlfriend communication - Girl Report, substance Use ARBA and Trauma History/PTSD-Girl Report, d) deleted Web measure, and 6) revised protocol and Initial Review to reflect amendment.

**Amendment #5,** approved on 10/30/2012, did the following: 1) Revised FUEL Curriculums: made formatting changes, added activities, and moved activities around to enhance program flow. 2) Revised Appendix P, 3) Revised recruitment flyer and letter to include the new recruiter's contact information. 4) Measure changes: a) added 5 items to the tracking and demographic measure; b) added 5 items to the observer evaluation forms; c) added facilitator feedback forms back in to the measure protocol; d) revised a few items to enhance participant comprehension.

**Amendment #6**, approved on 2/28/13, was a submission of revised security plan designed to require additional code words from the subject before the STI results are released via telephone in order to avoid additional breaches of privacy and confidentiality.

**Amendment #7**, approved on 3/18/2013, 1) Revised recruitment flyer and letter to include the new recruiter's contact information and the addition of the IMARA Cell number, and 2) Revised Appendix P.

**Amendment #8**, approved on 5/2/2013, 1) Addition of Release of Information form for daughters 18 years of age. 2) Revised Recruitment Letter and Recruitment Flyer to add the IMARA email address 3) Revised Protocol and Initial Application with regard to obtaining consent/assent, clarifying compensation, expanding recruitment efforts, and adding six items to the adolescent measures 4) Addition of Assessment Reminder Letter 5) Revised Appendix B to allow consent/assent to be read by participants themselves 6) Revised Eligibility Screening Questionnaire: Clinic Recruitment Sites to clarify compensation 7) Revised consent documents to clarify compensation 8)Addition of IMARA Informational Brochure 9) Addition of Eligibility Screening Questionnaire: Flyer/Brochure for potential participants referred by posted flyers/brochures 10) Revised Measures Protocol to add six items to the adolescent measure protocol and 11) Revised Participant Compensation Chart to add travel and/or parking payment for daughters coming to complete interviews without their mothers.

**Amendment #9,** approved on 5/20/2013, 1) Revised Recruitment Letter, Recruitment Flyer, and Assessment Reminder Letter, 2) Revised Measures Protocol to add one item 3) Revised Appendix P 4) Revised consent/assent documents to change compensation amounts in order to maximize acceptability of workshop interventions, compensate participants adequately for their time, and avoid recruiting a skewed sample of especially enthusiastic or altruistic participants 5) Revised Eligibility Screening Questionnaires to make changes in participant compensation amounts 6) Revised Participant Reimbursement Compensation Chart to make changes in participant compensation amounts 7) Revised Protocol and Initial Application to make changes in participant compensation amounts.

**Amendment #10,** approved on 7/9/2013, 1) Revised Protocol to describe procedure for how to address changes in compensation amounts for subjects who have already been consented; requested approval to pay the overall difference in compensation amounts, $30 for mothers and $20 for daughters, at the 12-month assessment.

**Amendment #11,** approved on 9/16/2013, 1) Revised Protocol to expand recruitment strategy to include girls who have mental health issues but are not seeking nor have ever sought mental health services, and to clarify eligibility requirements 2) Revised Appendix P to include new clinic liaison for Family Focus and to remove staff members no longer with study 3) New CBCL Recruitment Screener to be used to determine eligibility for those families who want to participate but have daughters who are not seeking nor have ever sought services 4) Revised brochure to clarify eligibility qualifications, update contact information, and edit design 5) Revised Initial Application to clarify that non-legal guardians who are primary caregivers can participate and to clarify that daughters who have mental health issues but are not seeking nor have ever sought services can participate. Includes a request for a waiver of consent because the recruiter will ask the primary female caregivers a series of screening questions to assess if families will qualify even if the daughters are not seeking nor have ever sought mental health services 6) Revised eligibility screening questionnaire for clinics to clarify that non-legal guardians who are primary caregivers can participate 7) Revised eligibility screening questionnaire for flyers to clarify when to use the CBCL screener and that non-legal guardians who are primary caregivers can participate 8) Revised Flyer to clarify eligibility requirements 9) Included DSMB Report 10) Revised Recruitment Script to include AA or Black participants 11) Revised consent documents to clarify that non-legal guardians who are primary caregivers can participate and to clarify that daughters who have mental health issues but are not seeking nor have ever sought services can participate, and to clarify the transportation reimbursement 13) Revised Recruitment Letter to include AA or Black participants and 14) Revised Appendix K to include another Family Focus clinic location in Englewood

**Amendment #12,** approved on 10/3/2013, requested permission to re-commence field assessments.

**Amendment #13,** approved on 11/15/2013, 1) Revised Protocol and Initial Application to include additional enrollment and STI verification steps and establish procedures for transporting subjects for research-related activities, and to add an additional question to verify that participants understand the consent/assent process 2) Revised consent and assent documents to clearly state the risks of receiving STI treatment if the subject tests positive, use the term “unique” instead of “anonymous” when referring to subjects’ STI number, and explain transportation procedures 3) Added an STI notification script and treatment plan 4) Added a Master List Verification Log for secondary independent verification of enrollment data, and 5) Added an STI Test Result Verification Log for secondary independent verification of STI test results. Our study conducted an internal audit of procedures and logs related to subject STI testing and reporting for this research, and no errors were found. The project director, data manager, and recruitment coordinator each independently reviewed and verified all logs related to STI testing and reporting for this research. A record of this verification process, including STI testing numbers, names, and STI results was created and signed and dated by the study PI, the project director, data manager, and recruitment coordinator. Our study has also adapted the recommended investigator tools (training and delegation logs) and will maintain these throughout the course of our study, 6) Revised Appendix P to include a new clinic liaison for Family Focus and to remove liaison for Chicago Youth Centers. Please note that we are keeping Chicago Youth Centers as a recruitment site and are in the process of identifying a new clinic liaison for that site 7) Revised Appendix K to include Rush University as a recruitment site.

**Amendment #14**, approved on 2/17/2014, 1) Revised Protocol and Initial Application to a) completely remove subject mental health as a screening criterion, b) expand recruitment techniques to use snowball sampling and utilize COIP Field Station staff, c) explain procedures if guardianship changes during the course of the study. We requested an expansion of the waiver of consent to release the new guardian’s contact information for these recruitment purposes, and d) clarify that unless both individuals request transportation together, for STI treatment we will only transport one individual (mother or daughter) at a time to preserve mothers and daughter's confidential test results. For workshop sessions, we will give mothers and daughters each the option of being picked up with other mother-daughter dyads since they will be participating in the intervention groups together and learn of each other’s involvement. If either member of a dyad states that she does not want to be transported with other dyads for the intervention groups, we will make alternate arrangements for their transportation, 2) Addition of a Legal Guardian Permission, Consent, and HIPAA Authorization document to be used for cases in which the legal guardian changes while the study is underway. This document clarifies that the new legal guardian is giving permission for the child to continue participation in the study, 3) Revised Measures to include a question asking participants if they heard about IMARA from someone who has participated in the program, 4) Revised Appendix P 5) Revised Youth Assent and Consent to broaden target population to include African American girls who are not seeking or receiving mental health services, 6) Revised Parental Permission, Consent, HIPAA Authorization to a) broaden target population to include African American girls who are not seeking or receiving mental health services and b) to clarify what will happen if mothers lose legal guardianship of daughters at follow-up, 7) Revised Flyer, Brochure, Eligibility Screening Questionnaire for Flyer/Brochure, Eligibility Screening Questionnaire for Clinic Recruitment Sites or Field Recruitment, Recruitment Script, and Recruitment Letter to broaden the target population to include African American girls who are not seeking or receiving mental health services, and clarify that participation in the study will not affect any treatment they may be receiving, 8) Added Eligibility Screening Questionnaire for Field Recruiters, 9) Revised IMARA Brochure wording, 10) Revised Eligibility Screening Questionnaires, with the addition of a question, “Is there anything that we should know about your child that would make it difficult for her to understand the study or participate in a group setting? For example, is she severely mentally ill or actively psychotic?”, 11) The protocol, initial application, and consent documents have all been revised to indicate that Dr. Stahl will have access to the UIC clinic records of those subjects who choose to be treated by her, 12) Revised consent documents so that statement about reporting STI results to the health department is only mentioned once and includes bold font and underlined text.

**Amendment #15**, approved on 4/4/2014, included the following changes:

1) Revised Appendix P 2) Revised STI Notification Script to clarify where STI results are reported.

**Amendment #16**, approved on 5/8/2014, included the following change:

Revised Protocol and Initial Application to change method of generating STI testing code numbers. A ‘Y’ will be used at the end of research numbers for adolescent youth samples, and an ‘M’ for mothers/female caregivers’ samples. Consistent with what our consent documents describe, these STI testing code numbers will still be unique for each individual participant, but this change will serve to reduce the potential for mistakes in linking STI Testing Code Numbers and Research Numbers.

**Amendment #17**, approved on 6/12/2014, included the following changes: 1) Notifying the IRB that we received approval from the NIH for an amended Certificate of Confidentiality to reflect changes in the scope to our study. These changes have all been approved by the UIC IRB in previous amendments, and 2) Revised consent documents to include the new email address for the PI, Dr. Geri Donenberg. Noted that her phone number listed in the consent documents has not changed, so participants with older versions of consent documents can still contact her by phone.

**Amendment #18,** approved on 8/7/2014, included the following changes: 1) Added question to Release of Information forms asking for school information, 2) Revised Appendix P to: a) remove Ruthie Moore and Mary Beth Tull, b)added names of five new staff, c) updated Erin Emerson and Brenikki Floyd’s email addresses, and 3) Revised recruitment documents to remove Ruthie Moore’s name.

**Amendment #19**, approved on 12/8/2014, included the following changes: 1) Revised Appendix P 2) Revised consent documents to indicate that a UIC adolescent medicine physician will be treating participants, and 3) Revised STI notification script to indicate that a UIC adolescent medicine physician will be treating participants.

**Amendment #20**, approved on 4/17/2015, included the following changes: added key research personnel.

**Amendment #21,** approved on 4/20/2015, included the following changes: Revised Protocol and Initial Application to remove CDISC from procedures and increase compensation amounts for baseline and workshops, revised consent documents to increase compensation amounts for baseline and workshops

**Amendment #22,** approved on 7/2/2015, included the following changes: added key research personnel, added Chicago Family Health Center as a research site and submission of revised eligibility screening questionnaires reflecting the increase in subject compensation previously approved in UIC Amendment #21.

**Amendment #23,** approved on 8/3/2015, included the following changes:Added a recruiter/facilitator and a faculty consultant, and revised format of STI Treatment Plan to make it easier to read.

**Amendment #24,** approved on 3/17/2016, included the following changes: Added a recruiter/assessor/facilitator, revised protocol and initial application to change format of baseline assessment and workshop day one, and describe data security encryption, revised consent documents to eliminate redundancies, make easier to read, and explain compensation and format changes for baseline and workshop day one, revised recruitment flyer to make more appealing, created IMARA website so that interested participants can access information about the study, revised workshop manuals to condense activities into a shorter timeframe, and revised participant compensation chart to reflect new format for baseline assessments and workshop day one and eliminated double payment for transportation reimbursement

**Amendment #25,** approved on 12/6/2016, included the following changes: 1) Added staff to Appendix P and removed staff.

**Amendment #26,** approved on 2/8/2017, included the following changes: 1) Removed Rush University as a recruitment site and 2) Removed staff from Appendix P

**Amendment #27**, approved on 1/26/18, notified OPRS/IRB of a change in the Principal Investigator's affiliation from the School of Public Health to the Department of Medicine in the College of Medicine; the investigator's campus contact information will otherwise remain the same.
