# Supplementary material for: IMARA: A mother-daughter group randomized controlled trial to reduce sexually transmitted infections in Black/African-American adolescents
Source: PLoS One. 2020 Nov 2;15(11):e0239650. doi: 10.1371/journal.pone.0239650 (PMC7605636; doi:10.1371/journal.pone.0239650)
Supplement: Attachment — Submitted filename: Response to Reviewers_R.docx [file pone.0239650.s005.docx]

April 27, 2020

Matthew Price
Academic Editor
PLOS ONE

Dear Dr. Price:

**Re: IMARA: A mother-daughter group randomized controlled trial to reduce sexually transmitted infections in Black/African-American adolescents (PONE-D-19-32194R1)**

Thank you for the opportunity to revise and resubmit for review the above referenced manuscript. We appreciate the additional helpful comments from the reviewer.

Below, please see the reviewer comments in **bold** and our responses in regular text. Changes in the manuscript are indicated by page and line numbers and they are tracked in the submitted manuscript. We sincerely hope these changes meet with your and the reviewers’ approval.

Sincerely,

Geri R. Donenberg, Ph.D.

Professor of Medicine and Psychology ⏐ University of Illinois at Chicago

Co-Vice Chair of Research ⏐ Department of Medicine

Director ⏐ Healthy Youths Program

Director ⏐ Center for Dissemination and Implementation Science

=========================================================================
**Reviewer Comments**

ABSTRACT

**The order of two sentences was a little confusing to me. You may wish to reword "Girls provided urine samples…” to something like "Girls provided urine samples to test for N. gonorrhoeae, C. trachomatis, and T. vaginalis infection at baseline and 12-months. Mother-daughter dyads were randomly assigned to IMARA (n=118) or a time-matched health promotion control program (n=81). Retention at 12-months was 86% with no difference across arms."**

The reordering of the sentence was done. Please see page 2, lines 33-35.

**You note that the intent to treat analysis was also significant “albeit with an attenuated magnitude”, yet in your results, the confidence intervals overlap. I suspect your study was not powered to see a difference between a 43% lower chance to contract an STI, and a 37% lower chance. I might change this to read that the ITT was "similar" to your main results, not "attenuated". Those two estimates are statistically very similar (and I would interpret them as essentially the same value).**

We changed to word “attenuated” to “similar”. Please see page 2, line 39.

METHODS

**Please do add the name of the test kits for CT/NG and TV. You don't need to add the sensitivity and specificity.**

We added the name of the test kits. Please see page 8, lines 163-164.

DISCUSSION

**In the discussion, "STI and HIV" is often mentioned together, yet your study does not test for HIV. You do note this in your response to one of the reviewers, but I think your discussion would benefit with a short mention of this in your 'limitations' paragraph so at least the reader is aware you're thinking of this, even if you weren't able to test for it.**

We added the following sentences to the limitations section of the manuscript:

“We did not test participants for HIV, because of the low incidence among adolescent girls. However, STIs are a proxy for unprotected sexual activity, a known risk for HIV.”

Please see page 20, lines 441-443.
